# Ins and Outs of Applied Behavior Analysis (ABA) Intervention in Promoting Social Communicative Abilities and Theory of Mind in Children and Adolescents with ASD: A Systematic Review

**DOI:** 10.3390/bs15060814

**Published:** 2025-06-13

**Authors:** Marco Esposito, Roberta Fadda, Orlando Ricciardi, Paolo Mirizzi, Monica Mazza, Marco Valenti

**Affiliations:** 1 Autism Research and Treatment Centre, Una Breccia Nel Muro, 00168 Rome, Italy; orlando.ricciardi@unabreccianelmuro.org; 2 Department of Applied Clinical Sciences and Biotechnology, University of L’Aquila, 67100 L’Aquila, Italy; monica.mazza@univaq.it (M.M.); marco.valenti@univaq.it (M.V.); 3 Department of Pedagogy, Psychology, Philosophy, University of Cagliari, 09100 Cagliari, Italy; robfadda@unica.it; 4 Department of Education, Psychology, Communication, University of Bari, 70121 Bari, Italy; paolomirizzi@yahoo.it; 5 Regional Centre for Autism, Abruzzo Region Health System, 67100 L’Aquila, Italy

**Keywords:** autism spectrum disorders, applied behavior analysis, social cognition, theory of mind, perspective-taking, false beliefs

## Abstract

Social-communicative abilities and theory of mind (ToM) are crucial for successful social interactions. The developmental trajectories of social and communicative skills characterizing individuals with Autism Spectrum Disorder (ASD) are rather complex and multidimensional, including components related to theory of mind. Due to its mentalistic nature, theory of mind has been rarely addressed as an outcome for Applied Behavior Analysis (ABA) intervention in children and adolescents with ASD. However, there is evidence that ABA intervention might be effective in promoting social abilities in individuals with ASD. Thus, this topic is worth investigating. We present a systematic review to explore the Ins and Outs of an ABA approach to promote social and communicative abilities and ToM in children and adolescents with ASD. We applied a PRISMA checklist to consider studies published up to December 2024. The keywords that we used were ToM, perspective-taking, false belief, social cognition, and mental states, in combination with ABA intervention and ASD (up to age 18). We searched for studies using Scopus, Google Scholar, and Medline. We included twenty studies on perspective-taking, identifying emotions, helping, detecting eye gazing, and social engagement, reviewing fifteen dedicated to teaching the interpretation of mental states (involving 49 children and 10 adolescents). The ToM was addressed with a multiple baseline design on target behaviors associated with ToM components such as identifying emotion, helping behaviors, and mental states. The intervention included a behavioral package consisting of Behavioral Skill Training, Derived Relations, video modeling, and role playing. The results indicated a significant number of participants who followed ABA intervention to promote social abilities and mastered the target behavior in ToM tasks; however, they showed maintenance and generalization issues across trials and settings. The role of predictors was highlighted. However, the studies are still rare and exhibit specific methodological limitations, as well as some clinical and ethical considerations. More research is needed to define best practices in ABA intervention to promote social abilities in individuals with ASD.

## 1. Introduction

Autism Spectrum Disorder (ASD) is a neurodevelopmental condition that emerges during the first years of life, characterized by impaired communication and social interaction skills, repetitive and stereotyped behaviors, activities, and interests, and hyper- or hypo-reactivity to sensory input or unusual interests in sensory aspects of the environment (Lord et al., 2018). The DSM-5 (the *Diagnostic and Statistical Manual of Mental Disorders, Fifth Edition*) emphasizes the heterogeneity and longitudinal nature of the individual characteristics, indicating how the levels of severity included in the diagnosis are dynamic and functional, and strictly related to the environment’s response to the individuals’ developmental needs (Hodges et al., 2020). People with ASD show different developmental trajectories, which might be influenced by some comorbidities: intellectual disability (ID), attention deficit hyperactivity disorder (ADHD), language disorders, and psychiatric disorders such as oppositional defiant disorder (ODD), obsessive–compulsive disorder (OCD), anxiety, depression, and schizophrenia (Crespi et al., 2010; Matson & Goldin, 2013; Matson & Shoemaker, 2009; Taurines et al., 2012). Studies have also found shared genes and genetic overlap between ASD and neurological/psychiatric comorbidities (Bishop, 2010; Gadow et al., 2014; Hossain et al., 2020). Language development may vary between and within individuals over time, involving different processes such as receptive and expressive language, syntax, semantics, and/or pragmatics. All these comorbidities should be considered when planning and evaluating the possible outcomes of the interventions aimed at promoting ToM abilities in individuals with ASD.

Applied Behavior Analysis (ABA) intervention is an effective, evidence-based practice with positive effects on individuals with ASD. A recent review evaluating the impact of randomized controlled trial (RCT) ABA intervention on promoting social skills in children with ASD indicated that there was significantly more improvement than eclectic interventions (Valenti et al., 2023). These results were confirmed by non-randomized studies, which reported that Early Intensive Behavior Intervention (EIBI) improved adaptive behavior, cognitive skills, expressive and receptive language, behavior, and socialization in children with ASD (Makrygianni et al., 2018; Reichow et al., 2012a). Similarly, Naturalistic Developmental Behavioral Interventions (NDBI) are effective in promoting social abilities in children and adolescents with ASD (Schreibman et al., 2015) compared to other types of interventions. Recently, a systematic review described a set of practices that have evidence of positive effects on autistic children and youth dependent (focused) on age and developmental area (Hume et al., 2021), such as Discrete Trial Training (DTT); Natural Environmental Teaching (NET); Pivotal Response Training (PRT); Cognitive Behaviorial Therapy (CBT); parent- and peer-mediated interventions; Antecedent-Based Interventions; behavioral momentum; Differential Reinforcement; Direct Instruction; Extinction; Functional Behavior Assessment; Functional Communication Training (FCT); prompting; video modeling; Reinforcement/Shaping; Self-Management; social narratives/scripting; schedules/visual aids; Task Analysis; Exercise; Picture Exchange Communication System (PECS); and Social Skill Training (SST). On the other hand, the long-term outcomes of comprehensive ABA-based interventions are the subject of debate. For children with autism, it is suggested that benefits in intellectual functioning and adaptive behavior can be sustained over time (Eckes et al., 2023). However, these studies also highlight that the effects may plateau or diminish without ongoing support, and improvements in core areas, such as language, may be limited or require continued intervention. Overall, while early intensive ABA can lead to enduring gains in certain developmental domains, the exact long-term outlook may vary depending on individual factors, the duration of intervention, and ongoing support.

### 1.1. Theory of Mind in People with ASD: Clinical Trajectories, Assessment, and Intervention

Theory of mind (ToM) is a complex social process that involves inferring the mental states of others. Recent studies have identified components such as cognitive ToM and affective ToM. The former involves attributing mental states (intentions, desires, and beliefs) to oneself and others, and using these attributions to understand, predict, and explain one’s behavior and that of others. The second concerns how individuals understand, predict, and explain their own and others’ emotions. From a theoretical perspective, cognitive and affective dimensions are partially interrelated (Goldstein & Winner, 2012). Specifically, affective ToM includes nine components hierarchically organized, such as recognition of emotional expressions and external causes of emotion, the role of desire, beliefs, external reminders of emotions, emotion regulation, displayed emotions, the role of moral dimension, and mixed emotions (Wellman, 2018). Two classes of behavior make explicit the role of the theory of mind: the first suggests that the individual has reached the awareness that other people have private experiences (mental states) different and unobservable of them, and the second that the child has acquired the ability to take the perspective of another animated persons/silhouettes. Competence in this regard has been observed in children between 2 and 3 years old with the emergence of simple emotion-based words, such as happy and sad. However, it is challenging to distinguish affective perspective-taking from related skills, such as empathy and a theory of mind. Additionally, ToM is generally distinguished into first- and second-order theories, where the first-order false-belief understanding develops around 4 years of age and concerns the ability to reflect on what another person thinks or feels, recognize that different people want different things and have different beliefs and knowledge, and understand a false belief. Second-order false beliefs involve predicting what one person may think of another and understanding lies, sarcasm, and metaphoric language, which are acquired between 6 and 10 years old (Xu et al., 2022).

Individuals with ASD display different developmental trajectories of ToM abilities and their precursors from the early years of life, showing differences in ToM abilities development. For example, children with ASD might show a low preference for human faces and eyes, focusing more on the movements of mouths and hands during conversations and less on stimuli that permit a more straightforward interpretation of social situations (Guillon et al., 2014). Similarly, individuals with ASD during a neuroimaging study on ToM show hypoactivation in these regions when performing tasks involving inference of mental states (Silani et al., 2008). In addition to social attention, two main cognitive functions can influence social processes: executive functions and language (Demetriou et al., 2019; Schwartz Offek & Segal, 2022). Even individuals who are fluent in a language may exhibit reduced abilities in natural settings. A research study explored the concept of compensation in ASD, where individuals exhibit good social skills despite underlying social-cognitive deficits (Livingston et al., 2019). The study focused on IQ, executive function, anxiety, and the relationship between social behavior and ToM performance involving 136 autistic adolescents who completed cognitive tasks and a self-report anxiety questionnaire, classified into High Compensators (good ADOS scores, poor ToM) and Low Compensators (poor ADOS scores, poor ToM). Findings showed that High Compensators demonstrated higher verbal IQ, better executive function, and greater self-reported anxiety than Low Compensators. Hence, the study highlighted the importance of cognitive assessments in understanding individual differences in ASD, where individuals can exhibit good social skills in structured tasks due to their high cognitive abilities. The study found that IQ, executive function, and anxiety play a role in compensatory mechanisms in ASD.

The measurement of social behavior typically relies on parent report questionnaires, like the Social Responsiveness Scale (Pruett et al., 2015), rather than diverse instruments based on single aspects using vignettes, such as the Sally-Anne task (first and second-order ToM), recognizing facial emotions, the Reading the Mind in the Eyes/Voice Test, and the Strange Stories, where participants identify double bluffs, persuasion, irony, and white lies (Benito-Ruiz et al., 2022). In the Reading the Mind in the Eyes Test, the participants respond via multiple choice, indicating an adjective that better matches the eyes shown, and the Advanced Theory of Mind Task (adaptation of Strange Stories) requires participants to describe a scene where the task requires the subject to go beyond the literal meaning of the text and make an inference about the story protagonist’s mental state in terms of persuasion, joking, sarcasm or a misunderstanding (Pino et al., 2020). Although stimulating the structured tasks based on the response to a single scene or discrimination between a few stimuli may not encounter cultural and verbal biases, as well as generalization issues and low social and ecological validity, the social skills laboratories could be a more accessible and more ecologically valid alternative to assess social ability. The Contextual Assessment of Social Skills (CASS) is an example of this, which measures conversations via two videotapes involving participants and two trained tutors, with the first videotape showing interest while the second shows boredom; individuals with ASD showed less engagement with an interested conversation partner compared to neurotypical peers (Simmons et al., 2021). The use of cartoons is also used; the Animated Theory of Mind Inventory for Children (ATOMIC), which comprises questions about characters’ cognitions and complex emotions (Beaumont & Sofronoff, 2008), involves a computerized test consisting of 18 cartoons depicting child, adolescent, and adult themes, each followed by two multiple-choice questions. The test was designed to assess theory of mind and central coherence abilities. The study’s results showed that children with Asperger syndrome performed more poorly on theory of mind questions compared to the controls, but performed equivalently on central coherence questions. The results also showed that children took longer to answer questions about ToM. The study found that children had difficulty inferring characters’ mental states, which was not due to remembering or integrating information. Even in this case, the social and ecological validity may be partial. Finally, Movie for the Assessment of Social Cognition (MASC) uses humor or sarcasm to provide a greater breadth of information about how much individuals with ASD respond to these stimuli. The disadvantage of these tests is that they often place excessive reliance on grammatical verbal skills and not on other aspects such as prosody or social cues (e.g., gestures, facial expressions, or gaze) that usually simultaneously accompany verbal utterances; participants must answer multiple-choice questions about the emotions, thoughts, or intentions of the protagonists in the movies. The test evaluates the understanding of non-verbal communication, irony, sarcasm, implicit social rules, blunders, and insinuations (Dziobek et al., 2006). There is an increasing demand for validated assessment tools with high ecological validity that simultaneously cover multiple dimensions of social cognition, including facial expressions, gaze, gestures, body language, pragmatic aspects of language (such as irony or sarcasm), and the interpretation of contextual clues. In natural circumstances, all of these aspects occur together, and their concurrent and immediate processing is required to interpret social behaviors appropriately.

Social and cognitive interventions for addressing the theory of mind in autism comprise two groups: focused ToM training or embedded in comprehensive social skills interventions. However, the interventions focused on ToM could result in scarcer generalization than comprehensives, which promise a more frequent transfer to natural settings (Bauminger, 2007; Begeer et al., 2011). The standard group interventions involving ToM are theoretically based on behavioral or cognitive behavioral principles, including didactic teaching and experiential practice, containing several learning sections and teaching strategies. For example, a group intervention about social interaction, including role playing, feedback, social games, social comprehension, and ToM abilities, was provided to 46 children and adolescents with high-functioning autism. The practical training comprises homework, parent training, and community activities to transfer the behaviors to real-life situations (MacKay et al., 2007). Likewise, the Program for the Education and Enrichment of Relationship Skills (PEERS^®^) is an evidence-based social skills program developed by the University of California, focusing on teaching skills for making and keeping friends and managing peer conflict and rejection. It is one of the few empirically supported social skills programs to disseminate published treatment manuals for mental health professionals and educators. Likewise, PEERS^®^ has been proven effective in improving social outcomes for adolescents with ASD and other social challenges like ADHD, anxiety, and depression, embedding evidence-based methods of treatment delivery of CBT, combined with the parent inclusion (Fatta et al., 2025). Group treatment is crucial for teaching social skills, as it allows for interactive learning with peers in a natural setting, involving up to ten adolescents at a time. The approach incorporates concrete rules and steps of social behavior, where adolescents enter conversations focused on their shared interests, commenting, asking questions, or offering compliments (Laugeson et al., 2014; Laugeson & Park, 2014). Additionally, role play demonstrations are a common technique where therapists model appropriate social behavior to simplify skills into concrete rules and steps. They also model inappropriate role play demonstrations, such as conversations that lack listening and watching, fail to take a turn, and stray off-topic. During conversations, adolescents learn to look for three concrete behaviors—verbal cues, eye contact, and body language—by receiving performance feedback from tutors. Treatment is generalized to other settings, such as reviewing homework assignments, where adolescents assess peer acceptance, and receiving successive homework reviews (Mausbach et al., 2010). Finally, parent involvement can help adolescents with behavioral rehearsals in natural settings and provide performance feedback, which may promote their learning. Parent groups occur weekly to teach social coaching techniques to their teens, and motivated teens agree to this coaching contract (Mandelberg et al., 2014). Nonetheless, the social group format has received a re-examination. A well-known study reviewed five social skill group interventions (lasting 5 to 20 weeks) for children and adolescents with ASD, providing evidence (Reichow et al., 2012b). Out of the five, four used a randomized wait-list control trial method, and one used a randomized controlled trial design with a no-treatment control. Another issue was that the participant had an IQ above the cut-off for ID, without a long-term outcome. In another more recent review (Miller et al., 2014), the authors noted that role play was identified as a standard and proper treatment technique. However, few social skill training programs focused on social cognition. Conversely, the article “Challenging Understandings of Theory of Mind” by Brownlow and O’Dell (2009) critically examines the dominant psychological theory that attributes the characteristics of autism to a deficit in ToM, focusing on what autistic people cannot do, rather than considering alternative explanations or strengths. Other groups, such as individuals with specific learning disabilities or even typically developing children with fewer siblings, can also perform poorly on ToM tasks. Furthermore, a significant proportion of autistic individuals perform well on such tasks, undermining the idea that ToM deficits are universal or exclusive to autism. Additionally, standard assessments may not capture the full range of social understanding or the diverse ways in which autistic individuals experience and express sociality. The importance of including the experiences and reflections of autistic individuals in research and theory development is recommended. They advocate for participatory approaches that recognize autistic people as experts in their own lives, challenging the dominance of non-autistic perspectives in constructing knowledge about autism. They argue for more inclusive, context-sensitive, and person-centered approaches that value autistic perspectives.

### 1.2. Behavior Intervention: Theoretical Framework and ToM Conceptualization

A behavioral intervention involves operant principles, as applied through analysis, to improve observable and measurable skills (Baer et al., 1987). The intervention focuses on changing the interaction between the child and the environment to provide learning opportunities. The first practices originating from the University of California included DTT as a structured model of the ABA curriculum, often embedded within other approaches, such as NET and verbal behavior teaching (Lerman et al., 2016). The goal is to teach the child to respond to language and social stimuli by repeatedly presenting a discriminative stimulus (SD), a structured prompt sequence, the target behavior, a reinforcer, and an intentionally short interval before the trial. Prompts are antecedent stimuli that increase the probability of correct responses in the presence of SD. They can be combined with the SD at the start of the discrete trial to ensure that a learner responds without error. They can also be delivered as part of an error correction procedure when the learner responds incorrectly, including most-to-least, least-to-most prompting, graduated guidance, and prompt delay (Cengher et al., 2016; McGhan & Lerman, 2013). Correct responses include social praise, access to a preferred item or activity (such as a token economy). Differential reinforcement is applied by not furnishing reinforcement to behavior under extinction. The educational curriculum can include various developmental skills, where therapists record the outcome of every learning trial, summarize it across blocks, and examine the data frequently (LeBlanc et al., 2020). A structured procedure such as DTT requires generalization sessions that focus on abilities that can be achieved using NET. In these sessions, technicians help children develop target skills by playing with them and using natural language and motivation when providing instruction. Natural teaching does not need sessions to generalize learned skills (Vivanti & Stahmer, 2021). Behavioral skills training (BST) is another effective method for teaching behavioral interventions to teachers, parents, and staff, including instructions, modeling, rehearsal, and feedback (DiGennaro Reed et al., 2018). BST is considered the gold standard for training others, but it can be expensive, time-consuming, and requires the availability of experts. Alternative training modalities, such as written manuals, videos, and computer-based instruction, have been developed to enhance efficiency, reduce costs, and increase availability. Studies show that video modeling with voiceovers can teach direct service staff to implement effective behavioral therapy. Additionally, videoconferencing (also known as telehealth or telemedicine) can enhance accessibility for staff and caregivers, allowing them to reach individuals who are far from qualified trainers. Moreover, it is a promising approach for teaching behavioral assessments and interventions (Esposito et al., 2020).

A recent comprehensive overview of perspective-taking literature from mainstream psychology and behavior analysis includes ABA and Relational Frame Theory (RFT) (Kavanagh et al., 2020). Traditional psychological research reported perspective-taking abilities divided into visual, emotional, and cognitive, with the first level involving detecting what others see only from that specific viewpoint, while the subsequent level involves understanding that even when two people can see the same object, they may do so from different points (Mazza et al., 2022). The Behavior Analytic Approach to Perspective-Taking theorizes that the theory of mind can be interpreted through a behavioral and operational approach, which helps overcome mentalistic definitions. Behavior analysts suggest that the ability to assume someone else’s perspective often interacts closely with environmental stimuli. This discrimination between stimuli may be related to perceptual dysregulation, a common issue in autistic individuals. The ability to attribute another person’s mental state involves observing environmental stimuli associated with the subject’s private events. Children learn to label and describe their private events by observing similar behaviors. Self-knowledge develops through shaping by the knowledge of others and social contingencies that reinforce the discrimination of one’s behavior. By asking questions such as “How are you feeling?”, other members of the verbal community shape an individual’s ability to discriminate their behavior, leading to better prediction and control over their behavior (Stewart, 2013). The primary definitions of identifying a mental state using behavioral concepts include Sidman’s equivalence relations, operant stimulus control, and inter-behavioral interpretation (Fryling et al., 2011; Schlinger, 2009; Sidman et al., 1989; Whalen & Schreibman, 2003). For example, according to RFT, verbal self-discrimination and perspective-taking are based on derived relational responding, distinguishing between non-arbitrary and arbitrary relational responding. Non-arbitrary relational responding involves relating one stimulus or event to another based on a shared physical property, such as shape, size, or color. It is directly acquired through contingencies and is highly developed in non-humans. In contrast, arbitrary or derived relational responding is more likely to be emergent regarding its acquisition; consequently, patterns include relations of coordination, distinction, opposition, comparison, hierarchy, and perspective-taking (Gibbs et al., 2024; O’Connor et al., 2017). Most empirical research on deictic relational responding (DRR) employed RFT, offering practice with three deictic relations (I–you, here–there, and now–then), as well as three levels of relational complexity, referred to as simple, reversed, and double-reversed relations (I–you/here) (Barnes-Holmes et al., 2004). Once established, these perspective-taking repertoires can generalize to new stimuli and natural conversation. Although deictic relations protocol offers valuable insights into perspective-taking in people with autism, it has been used extensively in empirical research with typically developing children (TDC) (Guinther, 2022), necessitating further investigations in clinical samples, along with meta-analyses (Diaz-Borda et al., 2024; Hempkin et al., 2024).

Concerns surrounding ABA, which point to normalizing client behavior to that of peers, have led some authors to analyze whether the abolitionist neurodiversity critique is ethically coherent and how behavior analysts should respond (Graber & Graber, 2023). First, they view autism as a natural variation in human neurocognitive functioning, not a disorder, while opposing interventions aimed at making autistic individuals appear more neurotypical. They draw a comparison to language instruction: while forcing a person to abandon their native language is wrong, helping someone learn a new language for practical benefits (if chosen freely) is not. They argue that ABA can be ethical if the goals are client-chosen, rather than imposed, and the methods are non-coercive and respect the individual’s identity. The benefits are meaningful to the client, not just to caregivers or society as a whole. Practitioners should adopt a client-centered ethic, ensuring goals and outcomes are aligned with the values and perspectives of the individual. Neurodiversity principles in ABA practice are both possible and necessary for enhancing autonomy, communication, and quality of life. Similarly, a recent scoping review maps emerging data on autistic well-being within a biopsychosocial context, excluding deficit-based research (Najeeb & Quadt, 2024). The review question focused on identifying biological, psychological, and social factors that contribute to well-being in autistic individuals within the neurodiversity-affirmative narrative, showing that biological factors contribute less to well-being than psychological and social factors. Additionally, nutritional practices and a diverse range of physical activities were identified as contributing to overall well-being. Improved sleep efficiency and management can improve well-being. Psychological factors were the most prevalently researched aspects of well-being. Receiving an autism diagnosis was identified as a factor that positively contributed to well-being, particularly in terms of propelling further progress with self-identity, along with self-acceptance, self-awareness, creative expression, and self-esteem, which were potentially protective against poor well-being. Finally, social support and social connectedness were the most prevalent social factors for well-being, as access to autism-friendly services and employment. Unfortunately, all studies display a gap in the literature on well-being research conducted in the autistic younger sample.

### 1.3. Scope and Research Questions

The study aimed to review the current literature concerning behavioral interventions (ABA and RFT approaches) on ToM in children and adolescents with ASD, providing insights for the appropriate clinical approaches in such developmental areas and raising methodological limitations to address. Therefore, we wanted to respond to the following research questions via PICO examples (Patient/Sample, Intervention/Alternative, Comparison, Outcome):

1. What is the state of the art regarding the outcome of ABA intervention on ToM in children and adolescents with ASD?
  - How have studies addressed maintenance and generalization issues of learning?
  - Which were the most adopted teaching strategies?
2. How have studies controlled experimental biases?
  - Research designs, assessment, and procedural integrity adopted;
  - Quality appraisal of proposals.
3. How should a clinician choose a ToM training for people with ASD?
  - Precursors and prerequisites;
  - Alternative to the intervention (comprehensive vs. focused)?

## 2. Materials and Methods

We followed the PRISMA checklist to conduct the review. Regarding the eligibility criteria, the articles were included for the current analysis if they met the following criteria: scientific articles were published in English in peer-reviewed journals; participants were children and adolescents (0–18 years) with a diagnosis of ASD (previously, pervasive disorders), which followed an intervention based on ABA or RFT. Studies involving other clinical populations have been excluded, as well as those that follow no behavioral-analytical interventions, such as CBT (including comprehensive or focused SST), neurodevelopmental, or psychological/psychoeducational training. Similarly, studies were excluded if they focused on narrative reviews, meta-analyses, or commentaries/proceedings. The study employed research designs that adhered to Cochrane’s definitions and criteria for randomized controlled and controlled clinical trials, including post-intervention and case studies (https://handbook-5-1.cochrane.org/chapter_6, accessed 12 December 2023). We reported all studies extracted in a table that contains the following information: ID, journal, DOI, title, abstract, and authors. Successively, two independent researchers (M.E and O.R.) assessed if each study had characteristics of eligibility (Yes/No), labeling motifs of exclusion (e.g., other languages, neuroscience, off-topic, theoretical/narrative, scoping review, systematic review, part in book, assessment, adults, treatment with medications, and correlational analysis, survey, and other clinical conditions), and, after having coded each study independently, counted the agreement (no occurred motifs of disagreement to discuss even if labeled exclusion criteria slightly differently) if such documents were available. Regarding the exclusion of qualitative studies, we aimed to analyze the variables that explain the variance in response to treatment, even if ABA intervention serves as a visual analysis and provides frequencies of mastery of the target. Concerning the information sources, two databases (PubMed and Google Scholar), a register (Scopus), and one reference list (previous reviews) were consulted to identify studies from 1987 to the end of December 2024. The search strategy for databases included the following filters ((theory of mind[Title/Abstract]) AND (autis*[Title/Abstract])) AND (behav*[Title/Abstract]) OR (((false belief[Title/Abstract]) AND (autis*[Title/Abstract])) AND (behav*[Title/Abstract])) OR (((perspective-taking[Title/Abstract]) AND (autis*[Title/Abstract])) AND (behav*[Title/Abstract])) OR (((social cognition[Title/Abstract]) AND (autis*[Title/Abstract])) AND (behav*[Title/Abstract])) and limits (peer reviewed scientific articles and in English). The algorithm was enriched with the following items: “mental state”, “desires”, “irony”, “social behav*”, along with the term “autis*” or “Asperger” in the title and abstract. A register of behavioral journals (e/ISSN) indexed in Scopus Elsevier with related editor (accessed date the end of December 2023) was extracted as follows: *Behavior Analyst* (07386729; Springer Nature), *Behavior Modification* (01454455; SAGE), *Behavior Research Methods* (1554351X; Spring-er Nature), *Behavior Therapy* (00057894; Elsevier), *Behavioral Interventions* (10720847; Wiley-Blackwell), *Behavioral Sciences* (e2076328X; MDPI), *Behaviour Change* (08134839; Cambridge University Press), *Behaviour Research and Therapy* (00057967; Elsevier), *Beyond Behavior* (10742956; SAGE), *Cognitive and Behavioral Practice* (10777229; Elsevier), *Cognitive Behaviour Therapy* (16506073; Taylor & Francis), *Current Opinion in Behavioral Sciences* (23521546; Elsevier), *European Journal of Behavior Analysis* (e2377729X; Taylor & Francis), *Journal of Applied Behavior Analysis* (00218855; Wiley-Blackwell), *Journal of Behavior Therapy and Experimental Psychiatry* (00057916; Elsevier), *Journal of Behavioral and Cognitive Therapy* (26663473; Elsevier), *Journal of Behavioral Education* (10530819; Springer Nature), *Journal of Behavioral Health Services and Research* (10943412, Springer Nature), *Journal of Positive Behavior Interventions* (10983007, SAGE), *Journal of Rational Emotive and Cognitive Behavior Therapy* (08949085; Springer Nature), *Journal of the Experimental Analysis of Behavior* (00225002; Wiley-Blackwell), *Journal of Verbal Learning and Verbal Behavior* (00225371; Elsevier), *Learning and Behavior* (15434494; Springer Nature), *Perspectives on Behavior Science* (25208969; Springer Nature), *Progress in behavior modification* (0099037X; SAGE), *The Journal of applied behavioral science* (00218863; SAGE).

**Figure 1:**
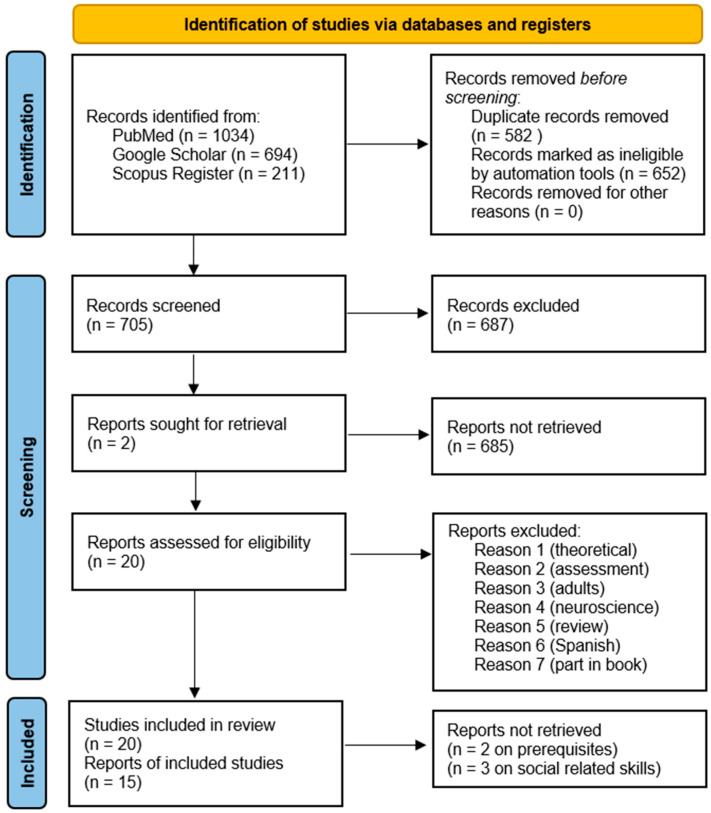
PRISMA flow diagram, which includes searches of databases and registers only.

We have reported the search strategy steps in Figure 1 via a PRISMA diagram illustrating the registers and papers included, retrieved, and reported, displaying several exclusion criteria, where two independent reviewers (O.R. and P.M.) completed the data collection process, reporting the following information for each study included in the review: country, number of participants, age of participants, diagnosis, assessment, comorbidities, target of intervention, clinical model, research design, interobserver agreement, baseline, post-probe, and follow-up. Furthermore, the reviewers assessed from 0 (absence) to 3 (exhaustive) the qualitative appraisal of the diverse studies (QuADS) on 13 items via a checklist regarding the quality of each section of the manuscripts: theoretical/background, aim/research questions, sample/participants, research design, assessment, appropriate data collection, justification for analytic method, data analysis, evidence, strengths and limitations (Harrison et al., 2021). An example of item 2 regarding the statement of research aim/s reports is as follows: 0 = No mention at all; 1 = Reference to what the sought to achieve embedded within the report but no explicit aims statement; 2 = Aims statement made but may only appear in the abstract or be lacking detail; 3 = Explicit and detailed statement of aim/s in the main body of report. This analysis of the quality described so far allowed for comparison of the methodological strengths and weaknesses of the studies considered. Additionally, the reviewers completed the Single-Case Experimental Design (SCED) Scale for each study to assess its risk of bias (Tate et al., 2008). The reviewers scored yes/no on 11 items, including clinical history, target behavior, research design, baseline, treatment phase, data recording, observer bias, independence of assessors, statistical analysis, replication, and generalization. For example, the first item asked if the clinical history of participants was specified by the authors (Age, Sex, Etiology, and Severity), while the last item required confirmation of whether the study reported evidence for generalization. The table compiles the issues identified in each study, along with the number of biases associated with each item. We calculated the agreement between raters on both instruments [agreements/(agreements + disagreements) × 100]. Finally, we report a qualitative synthesis of the study with a harvest plot to visually display the overall pattern of evidence and the direction of effects from different heterogeneous studies, where a standardized effect size was unavailable where input features include target behavior, the direction of the impact (positive, or no effect), comorbidity, the quality, and the risk of bias for each outcome across studies. Finally, to complement the statistical analyses, grayscale heatmaps were developed to display the distribution of generalization outcomes across ID, language skills, age, and teaching strategy. Boxplots were also produced to illustrate the distribution of increase percentages by the same variables.

## 3. Results

The 20 studies included varied in the target behavior of interventions, ranging from perspective-taking tasks (n = 10), identifying emotions (n = 3), helping/support (n = 2), prerequisites to detect eye/gazing and to label perceptions (n = 2), and social engagement/social-related skills (n = 3). The extraction table reports the following information: authors, year of publication, country, number and age of participants, comorbidity, target behavior, research design, and follow-up (Table 1).

### 3.1. Teaching Strategies

#### 3.1.1. Behavioral Packages

Generally, behavioral programs provide diverse ABA teaching strategies to teach the target behavior. For example, in one study on helping behavior (Harris et al., 1990), three adolescent boys (externally diagnosed) with rarely spontaneous speech received a stimulus preference/prompting/fading/reinforcement schedules package to transfer trained skills at the research office and home, including participation from their mothers and staff, for generalization to novel persons. The research design included sessions typically focused on baseline, training, and maintenance (lasting a few months). After each initial task, they were trained on two additional tasks, shifting to a multiple baseline across tasks rather than across participants. They required an 80% correct verbal response criterion for two days on each successive phase before training on the next task in the sequence. To ensure that the youths made a verbal offer of assistance, they had to repeat the phrase “Can I help you?” five times. A pool of 15 items consisted of screwing on a jar top, finding a quarter on the floor, inserting a key in a lock, putting tape in a tape recorder, opening a cabinet door, putting paper in an envelope, zipping a jacket worn by another person, and so on. At the baseline, the staff selected three tasks, showing each task three times a day for five days. Concerning training, the order of tasks was randomized. After meeting the criteria for each task, the youths moved ahead within their multiple baselines. Verbal prompts quickly faded as the youths complied. Maintenance procedures were identical to baseline, with the youth being thanked for assistance but not prompted to make such offers. Generalization was assessed for a new person in the training setting, a familiar person in the research office, and three novel tasks. Interobserver reliability was calculated. Regarding the results, the boys performed well during the maintenance but not in solving novel trials. In another study, researchers taught empathy to four children with autism who attended a behavioral program at the center and home (Schrandt et al., 2009). Specifically, the treatment package included modeling using audio scripts, physical guidance, affective discriminative stimulus compounds, prompt delay, behavioral rehearsals, and reinforcement in a pretend-play environment. Furthermore, they evaluated how empathy abilities transferred from training to probe stimuli in the absence of training and from training puppets and dolls to real individuals in a no-training environment. All participants had prerequisite skills in the vocal imitation of three-word phrases modeled by an instructor and on auditory recordings. Affective discriminative stimulus compounds contained one motor and one vocal component, presented in brief vignettes. These vignettes fell into three categories: sadness or pain, happiness or excitement, and frustration. Vignettes were randomly assigned. To promote generalization, dolls and puppets were not assigned to specific vignettes. Operationally, empathy was defined as a contextually appropriate reaction to an emotional presentation by a person, doll, or puppet that includes vocal and movement components and is displayed within 3 s. The participant had to pat the puppet’s arm and ask, “Are you okay?” For every category, the staff taught three vocal and one motor response. The responses taught for some vignettes could be used for any in the same response category, promoting generality to the training vignettes. The study consisted of four to five weekly sessions, each lasting 20 to 30 min, with trials lasting approximately 3 s. Participants viewed a vignette featuring a doll or puppet, and the prompter delivered either a consequence or no feedback. Three participants received training in only the sadness or pain response category, while others received training in other categories. In addition to dolls, puppets, and toys, a token economy system was discussed between the participant and the instructor. Participants chose from various preferred snacks and activities in exchange for ten tokens earned throughout the session. The prompter delivered one token per trial for appropriate sitting and attending to the vignettes. The study involved 30 training sessions, with seven training and three no-training probe-stimulus trials per response category. The prompter delivered manual and auditory prompts with a prompt–delay sequence (errorless). A behavioral rehearsal sequence was used for all training trials in the 0 s delay condition and when the participant did not respond or responded incorrectly in all other prompt–delay conditions. The prompter manually prompted a correct motor response and simultaneously played an auditory script on a Language Master. If the participant did not imitate the script, the prompter played the card again and waited for a response. If the participant did not respond, the prompter partially prompted the vocal and motor responses. If the participant did not respond or an error occurred, the sequence repeated until the participant emitted a correct independent response. The trials were randomly combined throughout all training sessions, incorporating vignettes similar to those used during training trials. The same procedures were in the baseline condition. The study was generalized to individuals with no prior training and other settings. The study used a multiple baseline design across participants to evaluate the effectiveness of the treatment package. The study examined responses to no-training probe stimuli, showing that appropriate responses generalized from training to no-training vignettes, dolls, and puppets for all participants. Other authors have used multiple exemplar training (MET) in the context of conditional discrimination to teach children with ASD the identification of what another person is watching (a prerequisite component of a perspective-taking repertoire) by following their facial orientation and eye gaze (Gould et al., 2011). The study involved three children, aged 3–5 years, who received an intensive home-based behavioral intervention program. The participants had to possess prerequisite skills such as the ability to sit and work at the table, visually discriminate between and tact all experimental photographs, and a history of successfully responding to visual prompts in the form of arrows. The study involved creating 24 stimulus cards, and the correct stimuli were four pictures of animals, vehicles, or colors. The cards were oriented to the left or right stimulus, as the conditional stimulus never included a person looking up or down, including it as a distractor. In the MET conditions, the cards included visual prompts, represented by red dotted arrows pointing from the person’s eyes to the stimulus they were looking at. The researcher implemented a concurrent multiple-probe design across participants with interobserver agreement (IOA). The study involved 24 randomized trials where participants received no prompting or feedback for correct or incorrect responses (baseline). Correct responses on trials of mastered tasks produced the child’s regularly programmed reinforcer. Training sessions consisted of 8–12 trials, with stimulus cards and therapist instructions identical to those in the baseline. The cards contained visual prompts, such as red dotted arrows drawn from the person’s eyes to the picture they were looking at. A most-to-least prompt fading procedure was used to fade out the visual prompts, with four levels of prompting. The prompt level was decreased contingent upon two consecutive sessions with 100% accuracy. If the participant responded incorrectly, the therapist proceeded to the next trial without providing feedback or administering reinforcement. The staff showed a generalization probe without prompting to determine if the mastery criterion occurred. If the response was 80% correct or higher, the intervention started with the next participant in the multiple baselines. If the correct response was lower than 80%, the training condition restarted with another set of stimulus cards. An error correction occurred in cases where the training procedure alone did not produce a favorable acquisition trend. When an incorrect response occurred, the therapist provided hand-over-hand guidance and modeled the correct vocal response, and then the next trial started. The study conducted generalization probes and natural environment probes to test for generalization to untrained stimuli. The procedure for natural environment probes involved a second person familiar with the task who was looking in one particular direction. The child had to name something directly in their line of vision, and the second person was required to rotate so that they were facing a different direction. During maintenance sessions, the staff did not provide prompting, and the consequences resulted in only brief praise from the therapist. During the training, the first participant responded at a 68% correct rate, failing to meet the criterion for generalization. The second participant achieved a natural environment probe at 62% accuracy. The last participant’s results showed no significant change in her behavior during the intervention phase until the error correction procedure occurred. Finally, two participants for whom follow-up data were available maintained skills up to 3 weeks post-intervention. Successively, a clinical study aimed to teach children with ASD a generalized repertoire of responding to disguised mands (non-literal requests), allowing them to respond to novel, untrained disguised mands (Najdowski et al., 2017). The study included MET, rules, role playing, and feedback involving three nine-year-old boys (two males) who had been receiving behavioral intervention. All participants communicated in full sentences and demonstrated repertoires of listener behavior (comprehension), rule-governed behavior, echoic mand, tact, and intraverbals. Sessions were conducted at home, including outside and at an amusement park. A correct response to a disguised mand involved initiating an action to give the speaker the item or outcome that was indirectly requested within 10 s of the disguised mand during baseline and within 3 s of the mand during the intervention, and completing the action within 30 s. Correct responses were converted to a percentage by dividing the total number of correct responses per session by the number of trials conducted and multiplying by 100. Two independent observers collected data. The study involved a 45 to 60 min daily session conducted 1 to 2 days per week. Baseline and post-training sessions consisted of 5 trials, while training sessions consisted of 10 trials. Five or seven different people presented disguised mand tasks across conditions to ensure generalization. A non-concurrent multiple baseline across participant design was implemented. In the training phase, disguised mands were introduced and randomly selected from a second list of 20 disguised mands. The therapist rotated the two exemplars semi-randomly during the 10-trial session, providing praise for correct responses within 3 s. If the participant failed to respond in 3 s or responded incorrectly, the therapist vocally modeled the correct answer. After mastery, a baseline probe included a novel and external person. Following post-training, participants responded accurately during the novel person probe and subsequent sessions. All participants generalized this ability, and one participant applied it to a novel community site. Another research study identified and responded appropriately to others’ preferences during play activities (Najdowski et al., 2018). The study involved three children with ASD, first (7-year-old girl), second (8-year-old boy), and third (5-year-old girl), who received behavioral intervention. The participants communicated using long sentences and had repertoires of listener behavior, including echoic responses, manding, gestures, intraverbals, and rule-governed behavior. They managed to choose during playtime with their peers, and parents were concerned that peers tended to lose interest in playing with their children. The study consisted of one or two 45 min daily sessions with peers and adults, held 1 to 4 days per week. Each session included two distinct assessment periods. The first assessment period assessed and taught participants to attend to the play chosen by their peers and to label their reactions to various toys. In each session, children sat with a play partner and six toys, which included a mix of toys the participant had previously nominated as either preferred or nonpreferred. The play partners engaged in scripted reactions to the presented toys, including indicators of interest (saying, “Yes” or “Sure, I love that game” or by beginning to play with the offered material) or disinterest (saying, “No” or “No, I do not like that game” or by engaging with an alternative toy). From a toy presentation and another, the participant and play partner engaged in cooperative play for 3 min with each toy or until the activity reached its natural ending. At the end of the session, the experimenter asked the play partner to leave the room and interviewed the children with post-play questions. If the participant did not respond with at least three items to each question, the experimenter prompted the participant to identify another toy. Training sessions were identical to the baseline, except for a unique set of six toys, the adults involved in training, and an intervention package consisting of rules, prompting, and feedback. The first assessment period involved the experimenter providing a rule: if a person played with a toy, they liked it, and if not, they did not. The experimenter provided prompting and praise for correct responses. The second assessment period involved the experimenter stating that sometimes a person will ask to play with something else. If the participant offered a preferred toy, received praise, and the children were permitted to play for up to 3 min, the experimenter would then proceed. If the participant did not offer a preferred toy, the rule was reminded, and the play partner continued to indicate wanting to play with something else every 3 min. After 80% accuracy, rules faded, and the skill reached mastery. Post-training involved generality probes with the same toys, experimenters, and procedures. Also, the staff conducted some natural environment probes without instructions or reinforcement. Correct trials involved offering a toy that the partner had played with, brought to the playdate, liked, or a novel toy (an IOA was assured). Results showed an improvement in learning, even with a novel person. Similarly, another study aimed to teach three children with ASD to tact (labeling) others’ private events using all five senses (Welsh et al., 2019). The research was conducted in the natural environment using MET to generalize to untrained stimuli. Children received ABA intervention using complete sentences and had prerequisite skills. The dependent variable was labeling the stimulus with which another person was interacting within 5 s when asked what the person could see, taste, feel, hear, or smell. The research design with IOA included non-concurrent multiple baselines across participants, and some sessions occurred in participants’ homes, each consisting of 10 trials. The trial involved MET, error correction, and reinforcement. Incorrect responses were corrected using a three-step procedure: a leading question, an experiential prompt, and a full vocal model. The first session involved the random rotation of two senses, followed by a probe with a novel person. After the training, the children showed an increase in stimulus identification accuracy. Finally, a study taught children with ASD and other developmental disabilities to respond appropriately to false-belief tasks, utilizing behavioral intervention strategies in natural environments with MET, prompting, and reinforcement (Dhadwal et al., 2021). Participants included three boys receiving ABA in center-based, preschool, and home settings. Three different false-belief tasks were used: the Sally-Anne task (digital cartoon), the Hide-and-Seek task (using actors), and the M&Ms task (using labeled containers). Response measurement involved recording correct or incorrect answers to questions associated with each task, with interobserver agreement (IOA) calculated to ensure reliability. A non-concurrent, multiple-baseline, across-participants design was used to evaluate the effects of MET, prompting, and reinforcement. Training sessions involved a least-to-most prompting hierarchy, MET with new stimuli, and reinforcement for correct responses. The Sally-Anne task was probed as a pre-test, after mastery of each trained task, and after post-training to identify generalization. The first boy met the mastery criterion for the hide-and-seek task within three sessions and the M&Ms task within two sessions and responded with 80% accuracy to the Sally-Anne post-test, the second met the mastery criterion for the hide-and-seek task within four sessions and the M&Ms task within three sessions and responded with 80% accuracy to the Sally-Anne post-test, and the third met the mastery criterion for the hide-and-seek task within seven sessions and the M&Ms task within three sessions and responded with 100% accuracy to the Sally-Anne post-test. All three participants learned the targeted false-belief tasks with generalization to untrained exemplars, people, and the cartoon version.

#### 3.1.2. Behavioral Skill Training

The behavioral skill training aims to teach sophisticated social and communication skills, such as being polite even if receiving an unwanted gift. Politeness implies being thankful toward the other person, refraining from explicitly expressing disappointment with the gift. This implicit social rule needs to be taught to participants to promote well-being in their daily social interactions. The study involved only two boys (5 and 9 years old) and one girl (7 years old), who were selected because their honest utterances were often misinterpreted as rude (Bergstrom et al., 2016). The participants communicated using complete sentences, employing mands, tact, and intraverbals, displayed rule-governed behavior, and learned through role playing. The training consisted of one to three one-trial sessions daily, followed by baseline and generalization sessions lasting less than 1 min, and training sessions lasting 5 to 10 min with IOA. Participants were required to express gratitude for an unwanted gift and be polite. Gift sessions involved an adult giving a child a wrapped gift with a nonpreferred or already owned item and asking them to share their thoughts. At appearance sessions, the adult arrived at home expressing satisfaction with their new appearance and asking what he thought. The altered stimuli included hair, pink clothing, baggy clothing, hats, eyeglasses, unusual shoes, and other items. The training procedures included providing descriptive rules, role playing, and corrective feedback to teach how to be polite when receiving an unwanted gift. The therapist praised correct responses that occurred within 3 s, provided a rule reminder and a model of the correct response, and offered feedback on missing elements. Likewise, participants received contingent feedback when they responded incorrectly or failed to respond within 3 s of the prompt. The training ended when correct responses to novel people and stimuli were elevated and stable. The results showed that the intervention was effective for all participants in using socially appropriate phrases to be grateful in this particular case of social interactions. These outcomes were quickly learned, and training resulted in generalization to untrained people and stimuli. The study tested for generalization across adults and stimuli but did not assess generalization across settings.

#### 3.1.3. Relational Frame Theory

Relational frame theory (RFT) provides a behavioral account of perspective-taking as DRR. People with autism scored similarly to the normative sample on questions requiring simple DRR but committed more errors when they reversed relations. Some studies have evaluated the use of deictic relational training in application with individuals with autism. However, one limitation in this growing area of research is that participants who demonstrated a transfer of function had mild forms of autism. One study targeted visual I–you deictic relations, which are the first to develop in individuals with autism (Belisle et al., 2016). The protocol Promoting the Emergence of Advanced Knowledge (PEAK) provides instructions for teaching deictic and other relational skills. Three boys with autism (18, 14, and 12 years old) were enrolled in a special school, exhibiting derived mutual and combinatorial entailment in tests of correspondence relations and mastery of simple “I” and “You”, but lacking single reversal “I” and “You” DRR. A multiple baseline design with multiple probes and IOA was applied, and the dependent variable was the percentage of correct responses. The study comprised an assessment of single reversal (You) (sYOU) and single reversal (I) (sI) associations for every stimulus combination during the baseline phase. In the sYOU relationship, the subject reported what he saw, while in the sI relation, the subject reported what the researcher saw. An image card was held vertically so that the subject and the assessor could see opposing sides to evaluate both relationships. The experimenter showed the participant the card on both sides. “If I were you and you were me, what would you see?” the experimenter posed as a test question to further explore the relationship. To assess the sI relation, the participant was given three seconds to respond to the question, “If I were you and you were me, what would I see?” before moving on to the next trial. The picture cards were also randomized inside each trial block. The participants received verbal praise from the experimenter if they answered the question correctly in three seconds. The experimenter gave the participant three seconds to provide a new response after stating, “Try again”, in response to an inaccurate response. The experimenter provided the individual with the correct response and proceeded to the next trial if they failed to respond correctly to this prompt. Verbal praise was not given. Each participant achieved 100% correct responses for the sYOU relation across three consecutive trials, and two demonstrated mastery of the sI relation. In the transfer phase, the participants demonstrated mastery of the untrained stimulus set. Another study aimed to extend the existing literature by demonstrating how to teach the identification of private events in others’ contexts using stimulus equivalence and transformation of stimulus function procedures (Schmick et al., 2018). The study involved three adolescent males who attended a special school (two were 13-years-old, and one was 17-years-old). They were aware of the non-arbitrary, cultural, and arbitrary relations of the RFT. Two sessions were conducted each week, each lasting 20 to 30 min. Materials referred to the PEAK-T program, Private Events of Others in Context, which included video-based scenarios for private events: happy (videos of a man crying at a wedding), angry (videos of a football player crying at a game), scared (videos of a man jumping away from a lake), and excited (videos of friends jumping around in a house). The dependent variable was the percentage of correct responses. Baseline and relational testing, with IOA verification, ensured that participants demonstrated correct responses prior to training. Tests for the AB relation involved presenting a video and asking what was happening; the BC relation involved asking, “If someone is (behavior in context), how might they feel?” Correct responses were reinforced with social praise, while incorrect responses received a prompt with the repetition of the correct response. Mastery criteria were determined as three consecutive trial blocks with a percentage of correct responses greater than 80%. Participants progressed to the relational testing phase once they had achieved mastery. Maintenance probes were conducted for all relations across all participants for two 2 week periods. Regarding the results, the first participant achieved mastery, while the other participants demonstrated infrequent maintenance. Another study enrolled five children aged 5 to 6 years and 1 month with ASD who participated in the study (Jackson et al., 2014). They had been diagnosed prior to the study, and at the study’s outset, they were administered the Childhood Autism Rating Scale (CARS). Their verbal comprehension skills were tested to ensure they could be enrolled in the study. They had all been undergoing ABA treatment and using a token economy system. Three of the children enrolled in the study completed the sessions at their homes, in a room with a table, two chairs on opposite sides of the table and the research materials, and distractions were minimized clearing the room; the other two children completed the sessions in a research room, with a table, two chairs on opposite sides of the table and the research materials. Each session lasted 30 to 60 min, and breaks were given approximately every 20 min. A multiple-probe design across participants was used. Three participants were randomly assigned to the experimental condition and the other two to the control group. Whereas the control group only completed four ToM probes and the initial Barnes-Holmes pre-tests once a week for four weeks, the experimental group completed deictic relations probes after receiving training for each level. A Multiple Stimulus without Replacement format preference assessment was conducted; the preferred item was made available to be exchanged for tokens, whereas the other items were used to reinforce on-task behavior. The procedures for the five different levels of perspective-taking varied, and the materials used included cards, boxes, dolls, pairs of items, a dollhouse, and containers. Interobserver agreement and procedural integrity scores were evaluated. During baseline sessions all participants completed pre-tests on all five levels of ToM and all three types of deictic relations (I–you, here–there, and now–then), across the three levels of complexity (simple, reversed, and double reversed), using a modified version of the Barnes-Holmes protocol, and were reinforced every 5 min regardless of the accuracy of their responses. Training on all three types of deictic relations frames (I–you, now–then, here–there) at the simple level of complexity was administered, reinforcing correct responses and giving feedback on incorrect ones, for eight trials per block. This was repeated until the participant responded correctly in 80% or more of the trials, at which time training stopped at that level and a mastery probe was conducted on simple deictic relations. If the participant could respond at 80% accuracy or better for the simple complexity probe, all levels of complexity on the Barnes-Holmes protocol and all ToM tasks were tested. Training then proceeded to the next level of complexity. All three participants in the experimental condition showed an increase in their scores on the Barnes-Holmes protocol using standard operant procedures. This did not seem to result in substantial or consistent increases in ToM scores for any of these participants. In general, the ToM scores of the control participants remained unchanged with repeated exposures throughout the study. Probes conducted throughout the study suggest that performance on each complexity level did not increase until the participants were directly trained. Despite increased performance on deictic relations, performance on ToM tests did not change significantly for any of the experimental participants. This suggests that training on the Barnes-Holmes protocol was insufficient to produce generalized changes in perspective-taking ability as measured by the ToM tests. The results of repeated ToM tests for the control participants demonstrate that this alone was insufficient to increase scores or levels passed consistently.

#### 3.1.4. Video Modeling

A study explored the efficacy of video modeling and reinforcement schedules for teaching perspective-taking to three children with ASD (7–13 years; males) and generalizing to novel stimuli (LeBlanc et al., 2003). The first participant had a mental age of 4 years and produced simple sentences when prompted; the second, with a mental age of 6, engaged in simple conversations about trains. The third boy, with a mental age of 13, read and wrote brief paragraphs. The children were seen twice to three times weekly at the special school, where they received three measures: Sally-Anne, M&Mst, and Hide and Seek. The study employed a multiple baseline design, with two tasks (M&M and hide and seek) for each participant, and the Sally-Anne task serving as both pre- and post-tests. The order of task training was counterbalanced across subjects, with each session lasting 4 to 10 min. The experimenter provided no feedback on performance, and after completion, the child received praise for effort and was allowed to choose the preferred item for participation. The child watched a video of an adult correctly completing a task, followed by a training session where the child responded to perspective-taking questions. Correct answers led to praise and preferred items or stickers. Incorrect responses resulted in replays and prompts for imitation. The training phase continued until a child correctly answered in the testing session on three consecutive trials. A follow-up was conducted one month after the final training session, with no video used before maintenance sessions. If a child failed a question, the authors provided a booster video modeling session to reassess the child. The study found that video modeling with reinforcement increased perspective-taking for the children. Despite consistently failing primary tasks and variants, all children eventually mastered the tasks and passed variants, even when novel vocal or motor responses were required. Follow-up evaluations were successful for two boys, with one requiring a booster session. The study also found that all participants failed the Sally-Anne pre-test, but two passed the task after intervention. A similar study employed video modeling to help acquire and generalize perspective-taking in three children with ASD (Charlop-Christy & Daneshvar, 2003). The children were older than four when assessed by the Peabody Picture Vocabulary Test (PPVT), showing a mental age of 2–5 years. A multiple-baseline design with IOA was used to assess the children, where multiple probes evaluated the generalization of untrained stimuli with balanced order. The measures comprised the Barney and Bugs Bunny task (pre- and post-test), while other tasks included a modified M&M test, hide-and-seek, tiger and zebra tasks, pizza tasks, and tiger false-belief tasks. The staff showed instructional videotapes (featuring adult models) to children to help them correctly answer all tasks, except for the Barney & Bugs Bunny task and its stimulus variations. The children were tested on the task twice during the baseline condition, and then video training for the first task began while the remaining tasks were still at the baseline. The experimenter reviewed the video with the child after viewing it, collecting data three times on the first task, with no indications concerning correct or incorrect responses. The participants followed close transfer tasks to test for generalization across stimuli. The procedure continued for all tasks, and once all tasks met the criteria, the post-test was administered. After completing training on all tasks, perspective-taking tasks without viewing a video were assessed to evaluate skill maintenance. The results showed that two participants gained on all tasks, exhibiting stimulus generalization and response generalization. At the same time, the third boy failed some tasks and the post-test, and his generalization was inconsistent during maintenance. Another study aimed at developing helping responses resulted in longer social interactions than other classes of social behavior. The study involved four children with autism who showed the imitation of movements and verbalizations and attended a private school where the training took place and was recorded. The sessions included video modeling with multiple exemplars (Reeve et al., 2007). Common parents were asked to define examples of their children helping, along with peers observed in classrooms during free-play activities for the same purpose. Subsequently, the following categories were reported: cleaning, replacing broken things, picking up items, organizing materials, positioning objects, carrying items, putting objects away, and setting up an activity. From these classes, the staff chose some activities to deliver opportunities for helping (e.g., tutor wiping something), where the verbal discriminative stimuli contained an exclamation (“oh”, “boy” “oops” and so on), contingently followed by a comment: “In what manner did this get disordered?”. The correct verbal component was always ‘May I help?’, emitted within 5 s of the initial exhibition of the SD. After offering help, the tutor replied with a simple “yes” or “sure”. The correct motor component was the child imitating the adult’s actions for the related activity. Therefore, a response was not counted as correct if it happened following video modeling or prompting. The study involved a 32-trial session consisting of 16 training trials, four within-category probes targeting a novel helping response within each child’s selected categories, two probe trials for categories where the child never received direct training, and 10 trials assessing no helping behavior. The intervention involved emitting nonverbal, verbal, and affective discriminative stimuli for the first helping activity programmed for the child and waiting for a maximum of 5 s for the child to emit the appropriate verbal and motor components of the helping response. The training procedure included a multiple baseline design, with the intervention introduced successively across participants. The study involved baseline trials without reinforcement, video models, or prompting, with trials terminated as they were in the baseline. Correct responses were immediately delivered during training trials, and if the child did not emit the correct helping response within 5 s, video models were then presented. Mastery was achieved with at least 94% accuracy in the first correct response presentation of training trials per session for four consecutive sessions. Additionally, the authors provided pre- and post-intervention trials regarding the generalization of behavior. The authors collected data by gathering the percentage of correct behavior and then monitored it 60 days after the experiment’s conclusion. After the training, each child learned to emit appropriate helping responses in the presence of specific discriminative stimuli during training trials. The generalization of helping responses occurred during probe trials. A follow-up session after 60 days revealed that helpful behavior had not changed; helping reactions also increased in the presence of novel and familiar cues, in a new environment, and with a new instructor. According to social validity criteria, the children’s helping behavior was suitable for their peers. A study which included various assessment tools on ToM (standard and DRR) investigated the response differences in three group of children examining the influence of prompting (Gómez-Becerra et al., 2007), involving TDC (5; 4–6 years), Down syndrome (5; 5–8), and ASD (5; 4–18), with their mental ages ranging from 4 to 7 years old (measured using the Wechsler Preschool and Primary Scale of Intelligence; WPPSI-III). An intrasubject design with comparisons was employed, focusing on interpreting mental and emotional states, DRR (symmetry, transitivity, and equivalence), discrimination skills for I/you and I/he-she, and discrimination skills for concepts of time and space. The dependent variables were measured using Sally-Anne, Emotion Faces, and the Pitu test. The Sally-Ann test inspired video modeling sessions, in which actors moved something from one hiding place to another while a hidden doll remained in the same location. Participants looked at the video with the least intrusive phase, and if they responded correctly to all test questions, the next phase was introduced. If they did not respond correctly, the next phase involved more intrusive prompts. The phases included prompts in which the narrator described the sequence and the actors talked among themselves and commented out loud on their actions. The results concerning Sally-Anne, Emotion Faces, and the Pitu test showed clear differences in responses from participants with ASD than controls (the level of derived relations was higher for the TDC than for any other participants). These findings also show deficits in perspective-taking skills by those with ID and language deficits. The children who scored best in tasks that contained skills considered prerequisites (I/you/he/she discriminations, spatial/temporal relationships, derived relations, and WIPPSI subscales) consequently scored best in the perspective-taking tests. All participants benefited from the procedures, scoring better in the perspective-taking tests after exposure to the feedback and prompt phases.

Another study has shown the efficacy of point-of-view video modeling (POVM) for teaching individuals to initiate and continue social interactions with others, including the maintenance and generalization of behaviors (Tetreault & Lerman, 2010). The research involved three children diagnosed with mild-moderate to severe autism, a boy of 5 years, another of 8 years, and a girl of 4 years who did not engage in spontaneous social initiations but could imitate three- to four-word sentences. The child’s language abilities and autism severity were assessed using the Preschool Language Scale, Fourth Edition (PLS-4), and the Childhood Autism Rating Scale (CARS). Training sessions comprised three sequences of social initiations such as the “Get Attention” script, which involved obtaining a conversant’s attention to display a creation made with a marker and a dry-erase board; the “Request Assistance” script, which taught a request for a conversant’s assistance in attaining and opening a clasped plastic box containing a bottle of bubble solution; and the “Share a Toy” script, which involved offering and requesting a Mr Potato head^®^ doll to a conversant and then requesting it back again. Post-viewing practice sessions instead consisted of exchanges, defined as eye contact and vocal behavior from the child that occurred before the vocal behavior of the conversant. The study employed multiple baselines across behaviors (scripts) design with IOA, with initial script assignment counterbalanced across participants; each participant began treatment on one of the three scripts while baseline occurred for the remaining two; the authors set generalizations to novel sets of materials throughout all baseline and treatment phases; hence, once a participant acquired the first intervened-upon script, treatment subsequently began. Therapists acted as conversants and were randomly assigned to all conditions and participants. At baseline, the children could exchange with the therapist without any contingencies for eye contact or vocal behavior. In the video-plus-food condition, one adult acts as the conversant, and a second acts as the trainer during video viewing and practice sessions. The participant was seated at the table with the DVD player and the video model for the target script. The trainer sat behind the participant during the video viewing and post-viewing practice sessions. During the video-plus-food condition, the girl began to orient towards the trainer instead of the conversant each time the conversant spoke, suggesting that conversant statements became discriminative for food reinforcers. In the least-to-most prompts phase, the trainer instituted a three-step least-to-most prompting procedure if a correct vocal response did not occur within 10 s of an opportunity during post-viewing practice sessions. In this phase, food items appeared if behavior occurred independently or with only a partial model; edibles did not occur in the case of a full model. For the “Share a Toy” script, no correct exchanges occurred during baseline, but increased once treatment started. Correct exchanges continued during the maintenance and follow-up sessions. In the generalization probes, no correct response occurred throughout treatment. In the third script, “Get Attention,” eye contact and vocal behavior increased simultaneously during intervention; however, there was little generalization to the novel materials. The boy did not engage in correct exchanges during the baseline, and there was no increase in correct exchanges during the video-plus-food condition. Across all phases, the boy’s exchanges during generalization probes remained at or below baseline levels. With the implementation of treatment for the “Request Assistance” script, correct exchanges gradually increased to mastery in 15 sessions and continued in maintenance, though generalization was limited. Eye contact increased during baseline for this script when the intervention began with the first script and was maintained with the introduction of the video-plus-food condition. However, during maintenance, eye contact and vocal behavior continued at approximately the same frequency. Similar increases in eye contact and vocal behavior occurred during the video-plus-food condition. The girl’s baseline response during the “Get Attention” script was at zero levels with little increase after 12 sessions in the video-plus-food condition. Both eye contact and scripted vocal behavior remained infrequent. The video-only phase started after a return to baseline; however, correct exchanges did not increase, and both eye contact and vocal behavior decreased. Similar results occurred for the “Request Assistance” script. Treatment began with the least-to-most-prompts condition, and correct exchanges reached mastery in five sessions. A food-only condition was introduced briefly, but behavior immediately decreased to one correct exchange. Correct exchanges were returned to mastery in two sessions and were maintained across three additional sessions during the conversant prompts condition. They were maintained when prompts were removed in the following phase. The girl’s baseline response during the “Get Attention” script was at zero levels, with little increase after 12 sessions in the video-plus-food condition. Both eye contact and scripted vocal behavior remained infrequent. To control this behavior, the video-only phase was initiated after a return to baseline; however, correct exchanges did not increase. The least-to-most-prompts condition was then implemented, and correct exchanges quickly increased with mastery in 10 sessions. However, an immediate decrease in exchanges occurred during a return to baseline. Similar results were obtained for the “Request Assistance” script. Treatment began with the least-to-most prompts condition, and correct exchanges reached mastery in five sessions. A food-only condition was briefly introduced, but behavior immediately decreased to one correct exchange. Correct exchanges were returned to mastery in two sessions and maintained across three additional sessions during the conversant prompts condition. The frequency of eye contact increased during baseline but decreased during the video-plus-food condition. Vocal behavior increased more rapidly than eye contact when least-to-most prompts were introduced. However, both behaviors occurred at approximately the same level during the conversant prompts and food-only conditions. There was limited generalization across all conditions. With the implementation of the least-to-most-prompts condition for the “Share a Toy” script, mastery was met in six sessions, and correct exchanges continued in the conversant-prompts and food-only conditions. The results showed that eye contact appeared to generalize across situations and was acquired and maintained more often than scripted vocal behavior.

Another brief report on identifying emotions with MET was conducted (McHugh et al., 2011). Three 5-year-old male preschool children with ASD, diagnosed by a clinician not associated with the study, participated in a home-based, intensive behavioral intervention program across multiple areas, such as self-help skills, communication, and toy play, but not emotion recognition. A multiple baseline design with IOA was conducted across four emotions (happy, sad, angry, and afraid) across participants, with maintenance and generalization probes across novel people, settings, and stimuli. Each session in baseline, intervention, generalization, and maintenance lasted approximately 2–5 min, and the sessions occurred ten times a day (six a week). Baseline sessions consisted of 12 instructional stimulus items, including video stories featuring familiar puppets. The training involved twelve video stories for each of the four types of situation-based emotions, where the instructor displayed the video and asked a question. The child’s response was recorded within 3 s, and correct responses were reinforced with verbal praise and reinforcing items. If an incorrect or non-response occurs, the instructor intervenes and implements a prompted learning trial to ensure a correct response. The first target response is introduced by conducting two or three mass trial prompts, followed by the second emotion (sad), and the third emotion (afraid). Once the child correctly identifies the first emotion, they are randomly alternated with already-mastered instructional videos until the correct response is 80% or above in two consecutive sessions. After mastery, the final step involves discrimination training between the target situation-based emotions and a previously trained situation-based emotion. The situation-based emotions are presented in unsystematic order, and the child must produce correct responses at 80% or above in two consecutive sessions. Four maintenance sessions were conducted 15 days after the generalization probes, each containing three stimulus items for each of the trained situation-based emotions. The outcomes established that children learned to tact emotions, generalizing to untrained stimulus items using a multi-component intervention (discrete trial instruction, prompt, error correction, and reinforcement).

#### 3.1.5. Social Skill Training

The present study (Plavnick et al., 2013) evaluated the effects of SST/ABA using video group instructions in four adolescents with ASD (13–16 years old; two males; mild to moderate intellectual disability), who were able to vocally request and comment for two sessions per week over 3 months. The study took place at school, where multiple probes across behavior designs with IOA were used to assess the effects of video group instructions (VGI) on three different social domains (social initiations, social awareness, and reciprocal social interactions). Researchers displayed three video clips, each lasting 20 to 30 s, depicting peers performing target behaviors. Before training, five baseline sessions, each lasting 20 to 30 min, were administered to each participant. During these sessions, five to seven opportunities to perform the target behaviors were provided, as the instructors engaged in antecedent behaviors to elicit the participants’ target behaviors. In particular, the instructor waited 10 s for the participant to perform the target response, refraining from providing explicit feedback or prompts. If the participant engaged in the target behavior, they were praised and received permission to join the social activity. During the 75 min social skills meeting, two 20 to 25 min VGI sessions were administered to teach target social behaviors; group meetings also included a review of rules, structured group activities, a snack break, and time to close the activity. The participants were instructed to watch the video and do and say exactly what the models did and said. If the participants engaged in the targeted response with the instructors as social partners, they received praise and permission to participate in the activity and interact with the social partner. If they failed to do so, the facilitator corrected the behavior, and they did not receive the aforementioned positive consequences. The group kept working on one social domain until 80% accuracy was achieved during the video modeling condition; video modeling then faded. Acquired skills were periodically probed to ensure maintenance. For social validity purposes, participants’ parents anonymously completed a satisfaction survey about the intervention’s goals, procedures, and outcomes: they all confirmed increased skills in maintaining conversations and starting social interactions at home. All participants demonstrated a major increase in the mean frequency of target behaviors in all social domains. However, participants may not generalize the acquisitions outside of the group context; future research should demonstrate generalization in other settings, such as schools. A more recent study aims to extend research on VGI by evaluating its potential for teaching adolescents with ASD or ID to respond to multiple social stimuli (Stauch et al., 2018). The study involved five adolescents diagnosed with ASD or ID via ADOS and Wechsler Abbreviated Scale of Intelligence (WASI-2) attending a special school. All participants received VGI within the same instructional group, which lasted four months, and included offering help, maintaining conversations, asking social questions, showing items to others, commenting on activities, and complimenting peers. Except for one student, all participants reached the mastery criterion for each behavior. A 17-year-old female with ASD had a lower standard score on the WASI-2. Another 16-year-old female with an ID had a borderline score on the WASI-2. A 16-year-old male diagnosed with ASD had a borderline score on the WASI-2. Another 15-year-old male with ASD had a score of 65. The last 17-year-old male with an ID reported a lower score on the WASI-2, speaking minimally, rarely making eye contact, and not typically interacting with peers or adults. He did not master the social skills targeted during the previous VGI. In addition to the five adolescents, two peers participated as social partners. In contrast, others created 13 video clips (ranging from 9 to 24 s) on extending or joining conversations and responding to affective behavior. The trial started when a peer engaged in the antecedent behavior designed to occasion the targeted social skill. The study found that the most effective target behavior was responding to the affective behavior of others based on nonvocal cues, such as body language and facial expressions. Participants were required to orient their eyes towards a peer, tact the private emotional event associated with observed affective behavior, and ask questions or comment about the target behavior. During baseline, participants received opportunities to perform each social behavior, and they were also given these opportunities during intervention. A multiple-probe design with IOA was implemented with a token economy system, rules, and a summary of the behavior taught. Baselines took approximately 30 min embedded within a 50 min social skills training session. The staff divided the participants into two groups and directed each group to join one of the typical peer groups. They played a group card game and engaged in an antecedent behavior. Depending on the successive targets, participants played a group game or activity while their peers were across the room. The participants did not receive immediate feedback during baseline probe sessions, meaning that there were no differential consequences for correct and incorrect responses. If participants performed a target response correctly, they experienced a naturally occurring social interaction with a peer that generally followed the specific target behavior. The intervention sessions were identical to baseline sessions, except for the addition of video modeling and error correction. The facilitator introduced the skill by making a statement that described the target behavior and instructed participants to watch the video. A video clip showed target behavior to participants, and the facilitator directed peers to provide one opportunity for each participant to perform it. The intervention condition included error correction following an incorrect response to ensure differential consequences for correct and incorrect responses. The study found that all participants, except one, demonstrated the acquisition of all three targeted behaviors. In contrast, the others demonstrated rapid skill acquisition, with some instances of generalization, suggesting that a natural setting, with peers as social partners, may enhance the generality of treatment effects. The last study examined the efficacy of SST on the ToM and social interactions in an 11-year-old student with ASD (Feng et al., 2008) who attended a general education program with an IQ of 85 measured by the Wechsler Intelligence Scale for Children–Third Edition (WISC) and a score of 79 on the PPVT. He exhibited inappropriate social interactions with adults and peers approximately one to five times during each 45 min class period. Three same-age peers with learning disabilities participated in training sessions with him, selected due to their high levels of socially appropriate behavior. The program included two units (ToM and social skills). Outcomes were assessed three times during baseline and at the end of each training session. Each evaluation consisted of six to seven items, each related to a situation-based scenario that taught the skill. All the scenarios reflected situations he would encounter in his daily interactions. Social interactions were collected through qualitative classroom observations in training and generalization settings. Each observational session lasted 40 min. In the training setting, observations of his social interactions took place during 1:1 and small-group instruction for the baseline condition and small-group training sessions for the intervention condition. In the generalization setting, his social interactions were recorded during 30 min lunchtime sessions and 10 min afternoon recess sessions (totaling 40 min per session). The ToM test was administered as a pre-test and repeated as a post-test. The ToM consists of 38 questions across eight real-life vignettes, representing content on situation-based emotion, desire-based emotion, belief, first-order false belief, second-order false belief, and fact, recall, or hint-type questions. The experimental design and procedures used a single-subject multiple-probe design across behaviors and settings. The baseline consisted of three evaluation probes for each targeted skill to obtain baseline learning outcomes. ToM and social skill training were conducted four times a week in the resource room, with each skill initially taught one-on-one with him. After achieving an 80% accuracy rate for three consecutive sessions, the training transitioned to a small-group setting with three preselected peers. After he performed at or above 80% accuracy on the learning outcome probes during three consecutive sessions of small-group training, the training began with another skill in the one-on-one setting. It was followed by small-group training on the same skill. The same training procedures were used during the small-group training sessions, with two exceptions: role plays were conducted with the selected peers instead of the trainer, and the training focused on students sharing their daily experiences rather than introducing stories. The maintenance, follow-up, and generalization phases included an assessment of the training program’s effectiveness in improving his social interactions. Also, interviews included teachers and three peers. Each interviewer responded to questions about changes in their social behaviors and peer interactions before and after the intervention, as well as their perceptions of the teaching materials, methods, and effects. The results suggest that the ToM and social skill training program improved the acquisition of ToM and social skills during the one-on-one small-group training, maintenance, and follow-up phases. After implementing the training program, his performance on the learning outcome evaluation probes improved dramatically, with the lowest point being 50%, exceeding all except the three baseline data points. High levels of performance occurred during the maintenance and follow-up conditions. The program used a systematic teaching procedure, including animated presentation of concepts, modeling, role plays, performance feedback, and experience connection, to teach important ToM skills such as identifying emotions, beliefs, and first- and second-order false beliefs.

### 3.2. Selection of Studies: Quality Appraisal and Bias

We selected 15 studies on traditional cognitive ToM tasks or that were strictly connected with understanding covert behaviors to interpret study outcomes, such as identifying emotion, helping behavior, and non-verbal communication, reporting their quality and bias assessment in Table 2 and Table 3. We removed two studies on eye gazing identification as a prerequisite and label experiences, and three on social initiation and reciprocity as consequences of other social skill trainings.

### 3.3. Report: Participants, Research Designs, Assessment, and Teaching Strategies

The study enrolled 49 participants, divided into small samples of three to five children or adolescents, for the training. Regarding the diagnosis and assessment, the studies report outside assessments, with two focusing on vocabulary and cognitive skills. A McNemar test on paired proportions (39 children and 10 adolescents out of the total sample) reported a respective difference of 94.7% (95% CI: 87–100%) and 85% (95% CI: 69–100%) regarding a positive outcome (*p* < 0.0001). The most comorbidities reported were moderate–severe ID in several studies, along with language impairment, compulsions, and challenging behaviors. The settings varied, including home, center, and special school. Single-case designs with IOA were reported in all studies that adopted a non-concurrent multiple baseline design (MBD) across trials (MBP) or participants (Kazdin, 2021). The methodology included a baseline assessment before the intervention and post-probes. Participants started baseline and training at different times; even a single module could begin at different times across participants. The subject is a case–control study, while the visual analysis of behavior permits a comparison of participants. Additionally, the prerequisite for experimental control is that the materials, modules, and individuals interacting with participants are randomly assigned. The behavior is scored as prompted, acquired, or not appropriate. Consequently, the experimenter compared pre- and post-training data and follow-up sessions. The dosage of the interventions ranged from 4 to 50 sessions, with an average of 35 sessions lasting slightly less than a month (20–35 days). The teaching strategies most commonly adopted were behavioral packages, including stimulus preference assessment, prompting, modeling, shaping, rules, feedback, and reinforcement schedules with tokens, along with video modeling, multiple examples, error correction, role playing, derived relations, social skill training, and behavioral skill training.

### 3.4. Report: Acquisition, Maintenance, Generalization, and Follow-Up

**Figure 2:**
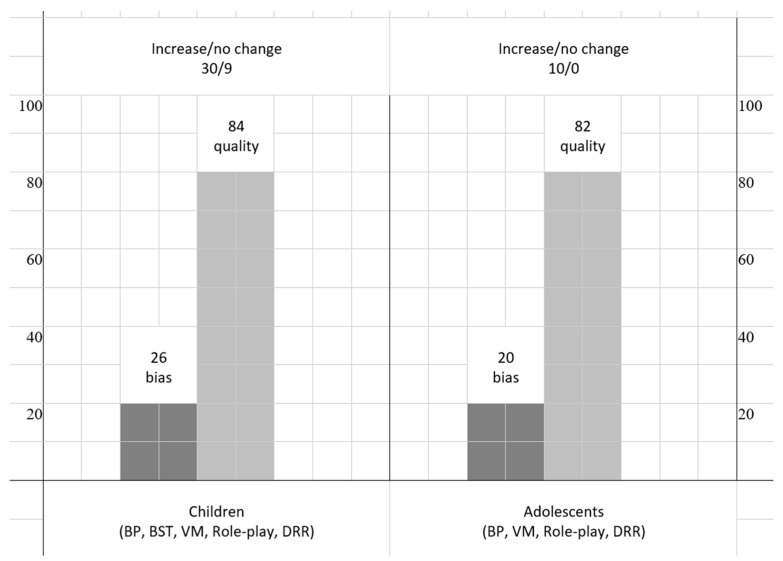
The harvest plot displays the number of increases and no changes, along with the related quality appraisal of studies and methodological bias. Note. Teaching strategies: BP (behavioral package), VM (video modeling), DRR (derived relational responding).

The studies to test acquisition followed a mastery criterion distinctive of an ABA program, which included two or three sessions where the target behavior occurred without prompt, along with continuous or discontinuous data reporting and procedural integrity (Han et al., 2023). The raw data on frequency were calculated using ratings or percentages to plot on a dispersion graph for visual analysis (composed from pre- and post-intervention phases). Out of fifteen studies, only four reported limited maintenance of the target behavior in their participants, eight showed bias in generalization regarding stimuli, environments, and trials, and three planned a follow-up study. Table 4 and Figure 2 present the selected studies, including the teaching strategies adopted and their corresponding outcomes. Analyzing a single component of the ABA program is limited to the broadly adopted behavioral package, or video modeling, the most adopted strategy.

### 3.5. Report: Precursors and Prerequisites

In narrative descriptions of the studies, several authors investigated the correlation between pre-session skills and outcomes, discovering that children with higher language levels also performed better in the derived relations acquired and consequently obtained better scores in the tasks regarding mental states; similar findings were evident in the case of more severe symptoms or cognitive impairments, in advance we show such investigation in all the studies. Starting with descriptive analyses, we identified eight studies, indicating that half of the participants had an ID (n = 7) or struggled with verbal communication (n = 11). However, this was often not specified, while the number of partially verbal participants was n = 4. Regarding the entire sample, the most frequently reported teaching methods were as follows: behavior package (n = 6), derived relations (n = 3), BST (n = 1), and video modeling (n = 5). Generalization was confirmed in 7 out of 15 studies. The mean of all studies about increase was 89.1% (three studies, respectively, 0%, 40%, and 67%) while the Effect of Teaching Method on the increase showed BP at 100% (one at 0%,), DRR at 66.7% (one at 0%), and one BST at 100%, while VM varied (40%, 67%, and 100%). The effect of ID on the increase varied (mostly 100%, one at 67%, one at 40%). Although most studies report high gains, the findings are not conclusive. The effect of language on the increase shows that those with low abilities can influence the outcome (67–40%) in contrast to those that were primarily verbal (100% and one null); however, partial language skills do not appear to impede improvement either. Teaching methods: six studies BP (100% increase), three studies on DRR (66.7%), one on BST (100%), and five on video modeling (84.5%). The behavioral program consistently shows 100% success, while video modeling yields high but slightly variable results, and DRR has mixed outcomes. Generalization was confirmed in 7 out of 15 studies, where BP was more likely to have entries (4 out of 5) than DRR and BST, both of which had generalization issues. Finally, VM displays mixed generalization outcomes—specifically, a gender breakdown. Most samples are male-dominated (often 100% male). One entry shows a higher proportion of females (66.7%), representing a 100% increase, which suggests that gender may not be a decisive factor in the outcome, although the sample size is small.

**Figure 3:**
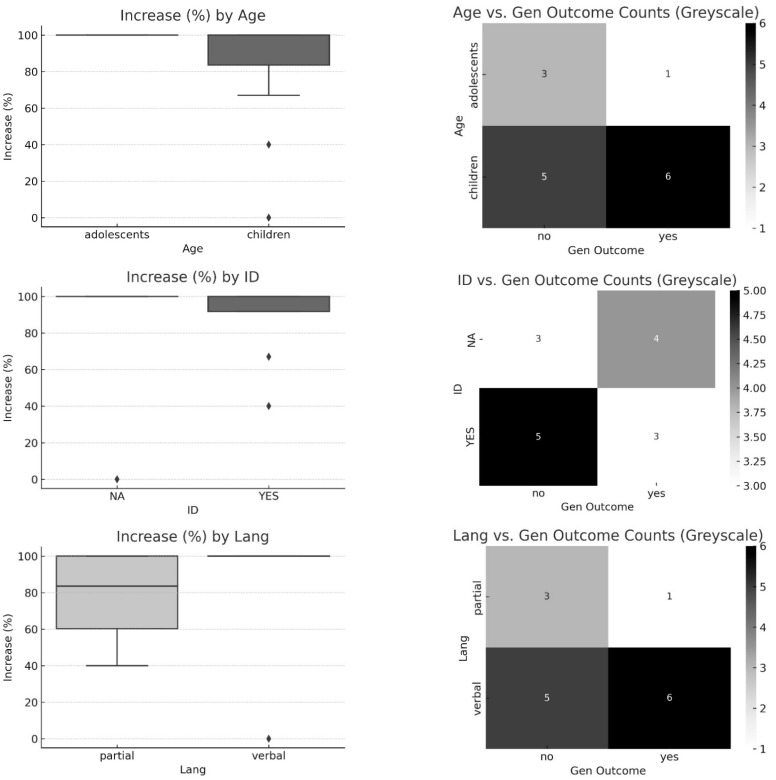

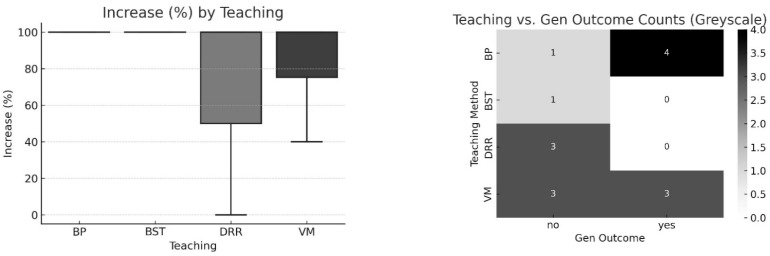
Boxplots and heatmaps of moderators. Note. Variables overview: ID (intellectual disability, YES/NA not available). Language type of language skill assessed: “partial” or “verbal”. Targeted age group: “children” or “adolescents”. Teaching method BP (behavioral package), DRR (derived relations), BST (behavioral skills training), VM (video modeling. Gen (generalization as dependent variable, yes/no). Male or female (number and percentage of participants). Increase (percentage of improvement/performance outcome as dependent variable). Moderator variables (potential): ID, Lang, Age, Teaching, Male, Female on 15 studies; most studies show high improvement (increase = 100), with few exceptions (e.g., one at 0%, another at 40%).

A detailed study of the impact of precursors is reported in Figure 3, which combines a boxplot and a heatmap for each variable, illustrating both increase and generalization. We report the results in vertical order of visualization, starting with the box plots. 

The first boxplot shows ID vs. increase. Both groups with and without ID have similar medians, with values near 100%. There is a slightly broader range in the ID group (some lower values observed, around 40–67%). However, there is no notable difference in performance improvement based on intellectual disability status, consistent with the Mann–Whitney U test result (*p* = 0.804). The second box displays language skills vs. increase. The verbal group shows a broader spread, including multiple studies at 100%, as well as a few lower outliers. The partial verbal group appears to have slightly less variability but a small sample size. There is no statistically significant difference (*p* = 0.162), but the greater spread in the verbal group might hint at variability worth monitoring in larger studies. The third box illustrates the relationship between age and increase. Both groups have medians close to 100%. The children show more variability, with several studies achieving 100% and a few falling below; there was no significant difference (*p* = 0.305). Though adolescents’ results are more consistent (likely due to a smaller sample size), the performance improvements appear broadly similar. The last box shows teaching methods vs. increase, where BP and VM methods show studies predominantly achieving 100% but with occasional lower outcomes (notably in VM and one in BP at 40%). DRR consistently achieved 100%. BST had one study at 100%. Moreover, a series of Fisher’s Exact Tests examined the relationships between study characteristics and the presence of generalization issues. Variables included intellectual disability status (ID), language skill (Lang), age group (Age), and teaching method (Teaching). The results indicated no statistically significant associations for any factor: ID: *p* = 0.619, Lang: *p* = 0.569, Age: *p* = 0.569, Teaching: *p* = 0.124. Despite the lack of statistical significance, a clear pattern emerged for teaching methods. On the other hand, we performed a statistical test studying the percentage improvement in performance using Mann–Whitney U tests for dichotomous predictors (ID, Lang, Age) and a Kruskal–Wallis H test for the multi-category predictor (Teaching). The results revealed no statistically significant differences (ID: *p* = 0.804; Lang: *p* = 0.162; Age: *p* = 0.305; Teaching: *p* = 0.503). However, descriptive trends were evident. Additionally, participants classified as verbal and those in the children’s age group exhibited greater variability in performance outcomes, suggesting that these groups might be more sensitive to differences in intervention approaches. The combination of non-parametric statistical tests and visual analytics proved valuable in detecting trends obscured by the modest sample size and limited distribution of study characteristics. Confirmatory studies with larger, more balanced datasets are necessary to substantiate these observations.

The first heatmap illustrates the relationship between the teaching method and generalization issues. Grayscale shading reflects count magnitude; darker shades indicate higher counts. Teaching methods include BP, BST, DRR, and VM. DRR had three studies reporting no generalization; BP had four studies, with only one without generalization; VM displayed a balanced distribution (three with and three without generalization); and BST resulted in one study. The second heatmap analyzes ID vs. generalization. Five studies with ID report no generalization than the other three. Studies that reported ID comorbidity were three, while the other four reported no generalization, suggesting no clear relationship between intellectual disability status and generalization issues based on this small dataset (the studies did not declare the comorbidity). The third heatmap displays language skill vs. generalization. The partial language skill group resulted in three studies, with no generalization and one “yes” response. In terms of verbal skill groups (presumed), there were five studies with no generalization and six that had generalization. A slightly higher number of verbal participants experienced generalization compared to those with partial skills; however, given the limited sample size, this observation is preliminary and requires further investigation, specifically regarding the relationship between age group and generalization. The last heatmap displays the frequency of generalization outcomes across different age groups, including adolescents and children. Adolescents were involved in three studies with no generalization, and one study reported a positive result. Children reported no generalization in five studies, while six confirmed it. More children encountered generalization than adolescents, although this could be due to sample imbalance (more studies on children than adolescents). Still, it may suggest age-related variability in how interventions generalize, which is worth exploring in larger studies.

## 4. Discussion

The state of the art regarding the outcome of ABA and RFT intervention on social cognition domains in children and adolescents with ASD has been investigated via the current review. All the studies covered social cognition domains or their consequences (social engagement), but only a subgroup considered theory of mind abilities as a target of the intervention. The results showed internal heterogeneity in response, even when the sample was reduced. The majority of participants mastered the target. However, the studies showed some limitations that need to be addressed. In particular, almost all the studies lacked generalization in natural contexts and follow-ups. Thus, the abilities were learned in highly structured settings, which were very artificial and reduced the ecological validity of the results. These limits were addressed through various strategies, including multiple examples and contexts, as well as, in some cases, peer interactions. Overall, the studies showed mixed results. Some studies attempt to explain the variability in results by considering prerequisites and highlighting the advantages of language abilities and symptom severity. Children with higher language levels easily mastered the target behaviors and maintained the skills acquired, albeit in a few training sessions. Likewise, a common bias of the studies resulted in scarce generalization because programs did not include the target in the natural environment. Regarding the training strategies, we observed that most studies employed fundamental behavioral strategies, while one-third incorporated other evidence-based practices, such as video modeling, group format, or role playing. The majority of the studies included tiny samples. Thus, due to the very low number of participants, it is not easy to analyze the effect of every strategy applied. Therefore, their effectiveness in teaching social skills still requires further investigation. As a future direction, it could be worth investigating the effectiveness of each component of the ABA program by manipulating the independent variable in a multiple baseline design, alternating the video modeling with imitation in vivo or other components (Riden et al., 2024). Exploring the advantages and disadvantages of the strategies adopted by studies, we noted that conditioning and the token economy system were the most common techniques. Such a technique is often associated with prompting, task analysis, shaping, and reinforcement schedules (https://www.bacb.com/wp-content/uploads/2020/05/RBT-2nd-Edition-Task-List_240830-a.pdf, accessed 29 March 2025). Besides operant conditioning, other evidence-based approaches were applied, such as video modeling. This technique allowed individuals to replicate the behavior displayed in the video or to discuss this behavior individually or in a group, including peers, to foster generalization. Another advantage of the research design adopted by the studies we considered is that it ensured procedural integrity, providing concurrent and nonconcurrent multiple baseline designs and reducing threats to internal validity (Slocum et al., 2022). The study’s analysis indicates some important guidelines for implementing ToM goals in ABA interventions. First, it is essential to identify the prerequisites that must be in place for ToM intervention to be successful. For example, comprehensive programs such as SST or CBT effectively integrate ToM modules. ABA approaches provide children and adolescents with opportunities to develop specific social cognition skills and engage in social initiatives. Symptom severity, cognitive, and adaptive skills need to be considered. Such an analysis of the requisites was lacking in all the studies considered. It is important to consider the great heterogeneity of the characteristics of individuals on the autism spectrum. This calls for a dimensional approach, in which individuals are not categorized into distinct groups. However, instead, all the individual differences are considered and taken into account to foster social abilities. Moreover, the sample needs to be more homogeneous in terms of prerequisites. Last but not least, staff should examine the social validity of that specific target behavior tailored to a person’s needs, since analysis of the cost-efficacy of training should be considered. The debate is still open. The interest in investigating ToM in older individuals with autism is growing (Fadda et al., 2024), indicating that adolescents and adults with ASD demonstrate weaker performance in ToM tasks compared to controls, mainly due to the characteristics of the tasks applied so far (Gao et al., 2023). These tasks reduce ToM abilities regarding a presence/absence phenomenon (Livingston et al., 2019). Thus, the strengths and the weaknesses of ToM abilities in individuals with ASD might still be unknown. This calls for a more dynamic, dimensional, and personalized approach, which needs to be embedded in ABA intervention to better promote complex social abilities in individuals with ASD throughout the lifespan. This also means that ABA intervention needs to listen to the voice of individuals with ASD. Listening to autistic people and developing goals and interventions based on functional and dynamic evaluation might help clinicians and researchers to understand that individuals with autism do not lack theory of mind overall, as a static characteristic. Instead, they develop many theory of mind competencies that must be considered along with language, cognitive, and general social development (Yu & Wellman, 2023).

ABA is a dynamic, evidence-based approach that continues to evolve, thanks to the research community’s ongoing address of various ethical, methodological, and clinical questions. In individuals with ASD, the perception of an experience as traumatic and its related traumatic stress is influenced by the epigenetic effects, which might act as a predisposition for Post Traumatic Stress Disorder, determining hyperreactivity to mild exposure (Kerns et al., 2015). In line with this consideration, an online survey was developed to investigate whether adults with autism perceived early intervention during childhood as a potential stressor (Kupferstein, 2018). A total of 460 respondents, including 243 autistic adults aged between 18 and 73 years and 217 caregivers, completed an online survey. Participants were recruited through social media networks, adult gatherings, social skills groups, and autism support groups nationwide. At least half of the participants were recruited via e-mail through the Interactive Autism Network (IAN) Research database and research registry. Adult inclusion criteria consisted of a diagnosis of autism or a self-diagnosis. Diagnostic reports were not collected, so the validity of the diagnosis was presumed. The survey included a 26-question questionnaire that combined both clinical and intervention-related questions. It was intended to be used only for this study, so it cannot be considered a clinical instrument. The results indicated that respondents of all ages who were exposed to ABA were 86 percent more likely to meet the PTSD criteria than respondents who were not exposed to ABA. However, since only the exposure to ABA intervention was considered in the study, an alternative explanation of the results might be that participants could have developed PSTS due to other unconsidered variables, like traumatic experiences at schools and/or in the community. Besides the underlined methodological issues of this study, including the recruiting criterion, the uncertainty of the diagnosis, and the fact that an online survey may have depersonalized sensitive information and decontextualized the meaning of the responses compared to contextually grounded conversation (Mishler, 1991), this study points to the importance of considering the potential stressful impact of early intervention in children with ASD.

Another study investigated the perception of 12 autistic adults of their early childhood experiences of ABA. In an interview, participants reported being stressed by repetitive tasks and physical interventions, feeling dehumanized and forced to perform, and experiencing a lack of empathy from the therapists (McGill & Robinson, 2020). Also, participants described negative impacts on mental health, including trauma, dependency based on fear, and the need to pretend to be someone else, leading to masking and camouflaging their identity. Of course, since this study is based on personal perception, the long-term outcomes might only be presumed. Also, since this is not a randomized controlled trial, but only a qualitative study without a control group, the causal role of ABA intervention in determining camouflaging and other negative consequences could also be explained due to other adverse experiences that the participants might have encountered in their life besides ABA intervention, like, as we said, before negative experiences at school and or in the community in general. Again, besides the methodological issues characterizing this study, it also highlights the need to consider the potential stressful impact of early interventions on vulnerable individuals, especially at a young age. Future research should also consider the subjective perception of well-being while involved in an intervention program or in long-term follow-up studies on the Ins and Outs of ABA intervention in individuals with ASD. Moreover, the intervention should be continually adapted to consider the responsiveness to the intervention and clients’ happiness and well-being (Leaf et al., 2022; Sandoval-Norton et al., 2019).

A possible way to foster subjective well-being in children, adolescents, and adults with ASD involved in ABA intervention might be to promote a deep knowledge of the heterogeneity of individuals with ASD among the practitioners. People certified in an RBT course (https://www.bacb.com/wp-content/uploads/2022/01/RBT-40-Hour-Training-Packet-240201-a.pdf, accessed on 16 May 2025) should continually enrich their knowledge of the wants and needs of their clients, not only when working with individuals with ASD, but also for all their clients in general. In line with the ethical and clinical issues highlighted by research (Rodriguez et al., 2023), the Behavior Analyst Certification Board (BACB) has updated its Ethics Code to emphasize compassionate practice and client well-being (www.bacb.com/ethics-information/ethics-codes, accessed on 16 May 2025), indicating that behavior analysts must provide effective treatment respecting clients wants and needs and caring for their dignity. Behavior analysts should offer a supportive environment and monitor the client’s willingness to engage in treatment.

## 5. Conclusions

The current review examined how ABA intervention has been applied to teach basic socio-communicative skills and ToM abilities in individuals with Autism Spectrum Disorder (ASD). Despite the numerous studies examining the impact of ABA intervention on various behaviors, the focus on social cognition and theory of mind remains relatively rare. The few studies that consider these interventions are heterogeneous, making it challenging to identify evidence-based practices that can be replicated in future studies. At the same time, this could be a promising line of research that warrants consideration. However, it is essential to consider that ABA intervention may promote early and non-verbal social abilities through operant conditioning, applied at various levels of complexity, ranging from discrete trial training to video modeling, role playing, and other methods. This might be of particular interest for individuals with ASD whose language and communication development might still be emerging. At the same time, ABA intervention has been proven to be effective also for individuals with ASD who are fluent in language. Thus, more studies are needed to understand for whom, for what, and in which conditions ABA intervention might be effective in fostering social-communicative abilities and ToM skills in individuals with Autism. In particular, it is essential to co-design future studies on theory of mind development with individuals on the spectrum, so that their perspective can be taken into account. Moreover, interventions should be situated in concrete contexts in which theory of mind abilities can help promote individuals’ well-being. Moreover, future interventions should consider the importance of teaching ToM abilities to caregivers and peers to enhance the general climate of daily natural environments. We strongly believe that such interventions could promote self-advocacy and well-being, thanks to better social skills.

## Figures and Tables

**Table 1:** Studies included in the systematic review.

Authors/Year	Country	Sample	Comorbidity	Age	Model	Target	Design	IOA	Follow-Up
Harris et al. (1990).	USA	3 (males)	ID	14–19	ABA	Helping	MBD+P	YES	NA
Schrandt et al. (2009).	USA	4 (3 male)	ID	4–8	ABA	Identifying emotion	MBD	YES	NA
Gould et al. (2011).	USA	3 (2 male)	NA	4–9	ABA	Detecting eye gazing	MBD concur.	YES	NA
Jackson et al. (2014).	USA	5 (males)	NA	5–6	ABA/RFT	Perspective-taking	MBD+P	YES	NA
Najdowski et al. (2017).	USA	3 (2 male)	NA	9	ABA	Responding to disguised mands	MBD	YES	NA
Dhadwal et al. (2021).	USA	3 (males)	DD	4–9	ABA	Perspective-taking	MBD	YES	NA
Najdowski et al. (2018).	USA	3 (1 male)	NA	5–8	ABA	Identify and respond to others’ preferences	MBD	YES	NA
Welsh et al. (2019).	USA	3 (1 male)	NA	6–8	ABA	to tact others’ private events	MBD	YES	NA
Bergstrom et al. (2016).	USA	3 (2 male)	NA	5	ABA/BST	Use socially appropriate lies	MBD	YES	NA
Belisle et al. (2016).	USA	3 (males)	ID	12–18	ABA/RFT	perspective-taking tasks	MBD+P	YES	NA
Schmick et al. (2018).	USA	3 (males)	NA	13–17	ABA/RFT	Identification of private events	MBD+P	YES	NA
LeBlanc et al. (2003).	USA	3 (males)	NA	7–13	ABA	Perspective-taking	MBD+P	YES	YES
Charlop-Christy and Daneshvar (2003).	USA	3 (males)	ID	6–9	ABA	Perspective-taking	MBD + whitin	YES	NA
Reeve et al. (2007).	USA	4 (3 male)	ID	5–6	ABA	Helping	MBD	YES	NA
Gómez-Becerra et al. (2007).	USA	15 ASD, TDC, DS	ID	4–8	ABA/RFT	Perspective-taking	Intra/intersubject	YES	NA
Tetreault and Lerman (2010).	USA	3 (2 male)	ID/Language	4–8	ABA	Social engagement	MBD	YES	YES
McHugh et al. (2011).	USA	3 (males)	NA	5	ABA	Recognizing emotion	MBD	YES	NA
Plavnick et al. (2013).	USA	4 (2 male)	ID/OCD	13–16	ABA/SST	Social engagement	MPDB	YES	NA
Stauch et al. (2018).	USA	5 (3 male)	ID	15–17	ABA/SST	Conversation	MPD	YES	NA
Feng et al. (2008).	USA	1 (male)	behavioral	11	ABA/SST	Perspective-taking	MPDB	YES	YES

Note. ASD: autism spectrum disorders; TDC: typical developmental children; DS: Down Syndrome; ID: intellectual disabilities; OCD: obsessive–compulsive disorder; ABA: applied behavior analysis; RFT: relational frame theory; SST: social skill training; MBD: multiple baselines across participant, MBD+P: across participant and probes, MPD: multiple probes, MPDB: multiple probe design across behavior; BST: behavioral skill training; IOA: interobserver agreement. NA: not applicable.

**Table 2:** Quality appraisal (QuASD).

Selected Studies	1. Background	2. Research Aim/s	3. Research Setting and Population	4. The Study Design	5. Appropriate Sampling	6. Rationale of Data Collection	7. The Format of the Data Collection Tool	8. Description of Data Collection Procedure	9. Recruitment Data Provided	10. Justification for the Analytic Method	11. The Method of Analysis	12. Evidence	13. Strengths and Limitations	Quality Scores
Harris et al. (1990)	1	3	3	3	2	0	3	3	1	2	3	3	1	2.2
Schrandt et al. (2009)	3	3	3	2	3	2	2	3	1	2	3	3	3	2.5
Najdowski et al. (2017)	3	3	3	3	1	2	3	3	3	2	3	3	3	2.7
Dhadwal et al. (2021)	3	3	3	3	1	2	3	3	3	2	3	3	3	2.7
Najdowski et al. (2018)	3	3	3	3	2	2	3	3	2	3	3	3	2	2.7
Bergstrom et al. (2016)	3	3	3	3	2	3	3	3	2	2	3	3	2	2.7
Belisle et al. (2016)	3	3	3	3	2	2	3	2	2	2	3	3	3	2.6
Schmick et al. (2018)	3	3	3	3	2	2	2	2	2	1	3	3	3	2.5
LeBlanc et al. (2003)	3	3	3	2	2	3	3	2	3	3	3	3	2	2.7
Charlop-Christy and Daneshvar (2003)	3	3	3	2	3	2	3	3	2	2	3	3	3	2.7
Reeve et al. (2007)	3	3	3	2	3	3	2	3	3	3	2	3	3	2.8
Gómez-Becerra et al. (2007)	3	3	3	2	3	3	2	2	3	3	3	3	3	2.8
McHugh et al. (2011)	3	3	3	2	3	2	2	2	2	3	2	3	3	2.5
Feng et al. (2008)	3	3	3	2	3	3	2	3	3	3	2	2	3	2.7
Jackson et al. (2014)	3	3	1	2	1	2	2	2	2	2	2	1	3	2.0
Quality Scores	2.9	3.0	2.9	2.5	2.2	2.2	2.5	2.6	2.3	2.3	2.7	2.8	2.7	2.6

Note. Items of QuASD: 1. Theoretical or conceptual underpinning to the research; 2. Statement of research aim/s; 3. Clear description of research setting and target population; 4. The study design is appropriate to address the stated research aim/s; 5. Appropriate sampling to address the research aim/s; 6. Rationale for choice of data collection tool/s; 7. The format and content of the data collection tool is appropriate to address the stated research aim/s; 8. Description of data collection procedure; 9. Recruitment data provided; 10. Justification for the analytic method selected; 11. The method of analysis was appropriate to answer the research aim/s; 12. Evidence that the research stakeholders have been considered in the research design or conduct; 13. Strengths and limitations critically discussed; general QUALITY. Example of Likert response from 0 to 3 on Background: 0 = No mention at all.; 1 = General reference to broad theories or concepts that frame the study., e.g., key concepts were identified in the introduction section; 2 = Identification of specific theories or concepts that frame the study and how these informed the work undertaken., e.g., key concepts were identified in the introduction section and applied to the study; 3 = Explicit discussion of the theories or concepts that inform the study, with application of the theory or concept evident through the design, materials and outcomes explored., e.g., key concepts were identified in the introduction section, and the application is apparent in each element of the study design. Interobserver agreement was reported as 80%.

**Table 3:** Bias assessment (SCED).

Selected Studies	1. Clinical History Was Specified. (Age, Sex, Etiology and Severity)	2. Target Behaviors. Precise and Repeatable Measures That Are Operationally Defined	3. Design 1: 3 Phases. The Study Must Be Either A-B-A or Multiple Baseline	4. Design 2: Baseline (Pre-Treatment Phase). Sufficient Sampling Was Conducted	5. Design 3: Treatment Phase. Sufficient Sampling Was Conducted	6. Design 4: Data Record. Raw Data Points Were Reported	7. Observer Bias: Inter-rater Reliability Was Established for at Least One Measure of Target Behavior	8. Independence of Assessors	9. Statistical Analysis	10. Replication: Either Across Subjects, Therapists or Settings	11. Evidence for Generalization	Bias (Items Failed)
Harris et al. (1990)	Y	Y	Y	Y	Y	Y	Y	Y	N	Y	N	2
Schrandt et al. (2009)	N	Y	Y	Y	Y	Y	Y	Y	N	Y	Y	2
Najdowski et al. (2017)	N	Y	Y	Y	Y	Y	Y	Y	N	Y	Y	2
Dhadwal et al. (2021)	N	Y	Y	Y	Y	Y	Y	Y	N	Y	Y	2
Najdowski et al. (2018)	N	Y	Y	Y	Y	Y	Y	Y	N	Y	N	3
Bergstrom et al. (2016)	N	Y	Y	Y	Y	Y	Y	Y	N	Y	N	3
Belisle et al. (2016)	N	Y	Y	Y	Y	Y	Y	Y	N	Y	Y	2
Schmick et al. (2018)	N	Y	Y	Y	Y	Y	Y	Y	N	Y	N	3
LeBlanc et al. (2003)	N	Y	Y	Y	Y	Y	Y	Y	N	N	N	4
Charlop-Christy and Daneshvar (2003)	Y	Y	Y	Y	Y	Y	Y	Y	N	N	N	3
Reeve et al. (2007)	N	Y	Y	Y	Y	Y	Y	Y	N	N	Y	3
Gómez-Becerra et al. (2007)	Y	Y	N	Y	Y	Y	Y	Y	Y	Y	N	2
McHugh et al. (2011)	N	Y	Y	Y	Y	Y	Y	Y	N	N	Y	3
Feng et al. (2008)	Y	Y	Y	Y	Y	Y	Y	Y	N	N	Y	2
Jackson et al. (2014)	N	Y	Y	Y	Y	Y	Y	Y	N	Y	N	3
Bias (items failed)	10	0	1	0	0	0	0	0	13	5	7	2.6

Note. IOA = 94% of agreement. Y: reported; N: not reported.

**Table 4:** Selected and reported papers.

Authors	ID	Assess.	Sample	Age	Model	Setting	Target	Design	Teaching	Increase	Gen.
Harris et al. (1990).	YES	Extern. +PPVT	3 (males)	14–19	ABA	home/school/office	helping	MBD+P	Prompting	3	− (trials)
Schrandt et al. (2009).	YES	Extern.	4 (3 male)	4–8	ABA	home/school	identifying emotion	MBD	P. Delay Behavior Rehearsal	4	+
Jackson et al. (2014).	NA	CARS	5 (males)	5–6	ABA/RFT	home	perspective-taking	MBD+P	DRR	0	− (trials)
Najdowski et al. (2017).	NA	Extern.	3 (2 male)	9	ABA	home/natural	respond to disguised mands	MBD	MET role play rules	3	+
Dhadwal et al. (2021).	YES	Extern.	3 (males)	4–9	ABA	center/home	perspective-taking	MBD	MET Prompting R+	3	+
Najdowski et al. (2018).	NA	Extern.	3 (1 male)	5–8	ABA	community	perspective-taking	Pre-Post	MET rules feedback	3	+
Bergstrom et al. (2016).	NA	Extern.	3 (2 male)	5	ABA	home	telling lies	MBD	BST	3	− (trials)
Belisle et al. (2016).	YES	Extern.	3 (males)	12–18	ABA/RFT	special school	relational responding	MBD+P	PEAK/ DRR	3	− (subjects)
Schmick et al. (2018).	NA	Extern.	3 (males)	13–17	ABA/RFT	special school	recognizing emotion	MBD+P	PEAK/ DRR	3 *	− (setting)
LeBlanc et al. (2003).	NA	Extern.	3 (males)	7–13	ABA	special school	perspective-taking	MBD+P	VM	3 *	− (setting)
Charlop-Christy & Daneshvar (2003).	YES	Extern. +PPVT	3 (males)	6–9	ABA	center	perspective-taking	MBD + whitin	VM	2 *	− (subjects)
Reeve et al. (2007).	YES	Extern.	4 (3 male)	5–6	ABA	special school	helping	MBD	VM package	4	+
Gómez-Becerra et al. (2007).	YES	WPPSI	15 ASD, TDC, DS	4–8	ABA/RFT	home/school/center	perspective-taking	Intra/intersubject	VM error corr.	2	− (setting)
McHugh et al. (2011).	NA	Extern.	3 (males)	5	ABA	home	recognizing emotion	MBD	VM/DTT Prompting error corr.	3	+
Feng et al. (2008).	NA	WISC	1 (male)	11	ABA/SST	school	perspective-taking	MPDB	VM role play feedback	1	+

Note. ID: intellectual disability (YES/Not Available); extern: received by external equipment, PPVT (Peabody Picture Vocabulary Test), WISC (Wechsler Intelligence Scale for Children), WPPSI (Wechsler Preschool and Primary Scale of Intelligence), CARS (Childhood Autism Rating Scale), Sample (number of participants and sex); ASD: autism spectrum disorders; TDC: typical developmental children; DS: Down Syndrome; Model (ABA: applied behavior analysis, RFT: relational frame theory, SST: social skill training); Design (MBD: multiple baselines across participant, MBD+P: multiple baseline across participant and probes, MPDB: multiple probe design across behavior); Teaching (P. Delay: prompt delay, PEAK: Promoting Emergence of Advanced Knowledge, DRR: derived relational responding, MET: multiple example training, R+: positive reinforcement; BST: behavioral skill training; VM: video modeling, error corr.: correctionGen: generalization (+ achieved; − partially achieved). * infrequent maintenance.

## Data Availability

We have shared all data in the manuscript, unless otherwise noted, as well as the original literature, which is available by emailing the corresponding author.
